# Disrupting CDK9 activity suppresses triple-negative breast cancer and is enhanced by EGFR Inhibition

**DOI:** 10.1007/s13402-025-01154-6

**Published:** 2026-01-08

**Authors:** Vera E. van der Noord, Ronan P. McLaughlin, Jessica S. Karuntu, Jichao He, A. Mieke Timmermans, Sunita K. C. Basnet, Yi Long, Sarah Al Haj Diab, Solomon Tadesse, Natalie Proost, Bastiaan van Gerwen, Bjørn Siteur, Marieke van de Ven, Chantal Pont, Sylvia E. Le Dévédec, John W. M. Martens, Shudong Wang, Yinghui Zhang, Bob van de Water

**Affiliations:** 1 Division of Cell Systems and Drug Safety, Leiden Academic Centre for Drug Research, Leiden University, Einsteinweg 55, 2333 CC, Leiden, 2300 RA The Netherlands; 2 Department of Medical Oncology, Erasmus MC Cancer Institute, Cancer Genomic Netherlands, Erasmus University Medical Center, Rotterdam, 3000 CA The Netherlands; 3 Drug Discovery and Development, Clinical and Health Sciences, University of South Australia, Adelaide, South Australia 5000 Australia; 4 Preclinical Intervention Unit, Netherlands Cancer Institute, Mouse Clinic for Cancer and Aging (MCCA), Amsterdam, 1066 CX The Netherlands

**Keywords:** Triple-negative breast cancer, Transcriptional dependencies, CDK9, EGFR, Combination therapy

## Abstract

**Purpose:**

CDK9, in complex with cyclin T1 or T2, is essential for mRNA transcription by enabling paused RNA polymerase II to proceed into elongation. Increasing evidence highlights CDK9’s involvement in transcriptional addiction in cancer. Triple-negative breast cancer (TNBC) is an aggressive breast cancer subtype for which effective targeted therapies remain limited. Here, we aimed to define the therapeutic potential of novel CDK9 inhibitors in TNBC.

**Methods:**

We explored the efficacy and mechanism of action of novel CDK9 inhibitors, alone or in combination with EGFR inhibitors, using TNBC cell lines and in vivo xenograft models.

**Results:**

Targeting CDK9 significantly impaired proliferation and induced apoptosis in multiple TNBC cell lines. Transcriptomic analyses revealed that CDK9 inhibitors induced downregulation of genes involved in transcription, cell cycle progression, and oncogenic signalling pathways, including TGF-β and Wnt/β-catenin signalling. Combined CDK9 and EGFR inhibition disrupted transcriptional programs, enhanced TNBC cell death in vitro, and acted synergistically to reduce tumour growth in PDX and Hs578T xenograft models, although this combination was also associated with increased toxicity.

**Conclusion:**

Our results position CDK9 as a promising therapeutic target in TNBC, either alone or in combination with EGFR inhibition, provided that side effects associated with this combination treatment can be controlled.

**Supplementary Information:**

The online version contains supplementary material available at 10.1007/s13402-025-01154-6.

## Clinical trial number

Not applicable.

## Introduction

Triple-negative breast cancer (TNBC) is a particularly aggressive subtype of breast cancer characterized by the absence of estrogen receptor (ER), progesterone receptor (PR), and human epidermal growth factor receptor 2 (HER2) expression, which accounts for approximately 15% of all breast cancer cases [1, 2]. Although novel therapies, including PARP inhibitors and immune checkpoint inhibitors, have slightly improved the prognosis for some TNBC patients compared to neoadjuvant chemotherapy and surgery alone, the overall prognosis for TNBC remains poorer than for other breast cancer subtypes [2–4]. This highlights the urgent need to develop effective therapies that specifically target TNBC-related weaknesses. However, efforts to target other TNBC-specific aberrations, including EGFR, have not yet been translated into clinical benefit beyond standard therapies [4, 5].

Genetic aberrations (e.g. EGFR and MYC amplifications) in cancer cells also lead to dysregulated transcriptional programs which render cancer cells highly dependent upon exceptionally high rates of transcription of genes critical to promoting proliferation [6–8]. The expression of certain oncogenes and short-lived pro-survival genes is exquisitely sensitive to disruption of the complexes which regulate transcription [9, 10]. Consequently, targeting transcriptional addiction has emerged as an important therapeutic strategy [7]. Rather than targeting pharmacologically intractable, disease-driving oncogenes such as MYC, attention has turned to the complexes which govern mRNA transcription in cancer cells. To this end, blocking the activity of a subgroup of cyclin-dependent kinases (CDKs), which are critical for productive mRNA transcription, may represent a valuable tool in combating TNBC [11].

CDK9 and its regulatory cyclins, cyclin T1 or T2, form the positive transcription elongation factor b (P-TEFb) complex [12, 13]. P-TEFb, in conjunction with CDK7/cyclin H/MAT1, is indispensable for ensuring transcriptional initiation and overcoming promoter-proximal pausing of RNA Polymerase II (RNAPII). Phosphorylation at Ser5 by CDK7 of RNAPII’s C-terminal domain (CTD), the catalytic component of master transcription factor TFIIH, initiates transcription but also causes recruitment of negative elongation factors (e.g. NELF and DSIF) which pause RNAPII during this process [14, 15]. CDK9 is recruited by BRD4 and induces pause-release by phosphorylating NELF and DSIF [12, 13]. Moreover, CDK9 facilitates transcription elongation by phosphorylating RNAPII CTD at Ser2.

Despite playing critical roles in general transcription, inhibition of CDK7 and BRD4 have been implicated in preferential downregulation of cancer-related genes, such as MYC, thereby selectively killing cancer cells [16–18]. Recent data have demonstrated that also broad-spectrum CDK inhibitors with potent activity against CDK9 are effective at inhibiting the growth of cancer cells, including breast cancer [19–22]. While the impact of CDK9 on gene regulation has been explored to a lesser extent, emerging reports suggest CDK9 is crucial to the expression of pro-survival genes and maintaining stem-cell-like features of cancer cells [23–27]. Moreover, its inhibition could prevent drug resistance against a variety of inhibitors [23, 25, 28]. We have previously demonstrated that a Cdc7/CDK9 inhibitor synergizes with EGFR inhibitors in TNBC, and that synergy between multiple CDK9 or CDK12/13 inhibitors and kinase inhibitors, including EGFR inhibitors, is mediated through the inhibition of ABCG2 [29, 30]. Collectively, this underscores the potential of targeting master transcriptional regulators, such as P-TEFb/CDK9, as a robust strategy for various cancer types. However, these insights primarily stem from in vitro studies, the use of broad-spectrum CDK inhibitors and/or models of (hematologic) malignancies other than TNBC. Studies investigating the impact of selective CDK9 inhibitors in TNBC models, both in vitro and in vivo, are limited [18]. Moreover, to date, no selective CDK9 inhibitor has reached clinical approval, underscoring the need for the development of new, selective CDK9 inhibitors.

In this study, we revealed the efficacy of a new panel of potent CDK9 inhibitors in TNBC, both in vitro and in vivo. Targeting CDK9 effectively disrupted key oncogenic pathways, and significantly reduced the expression of critical transcription factors. Silencing these transcription factors using RNAi demonstrated that TNBC cells rely on them for survival, highlighting them as novel therapeutic targets indirectly affected by CDK9 inhibitors. Moreover, we show that EGFR inhibitors could synergistically enhance the effects of CDK9 inhibitors, leading to further transcriptional dysregulation. This combination therapy reduced tumour growth in vivo, however, it also caused weight loss in mice, highlighting the necessity for further investigation to better understand and mitigate potential side effects associated with this treatment approach.

## Results

### CDK9 expression correlates with MCL1 expression in TNBC tumour tissue

**Fig. 1:**
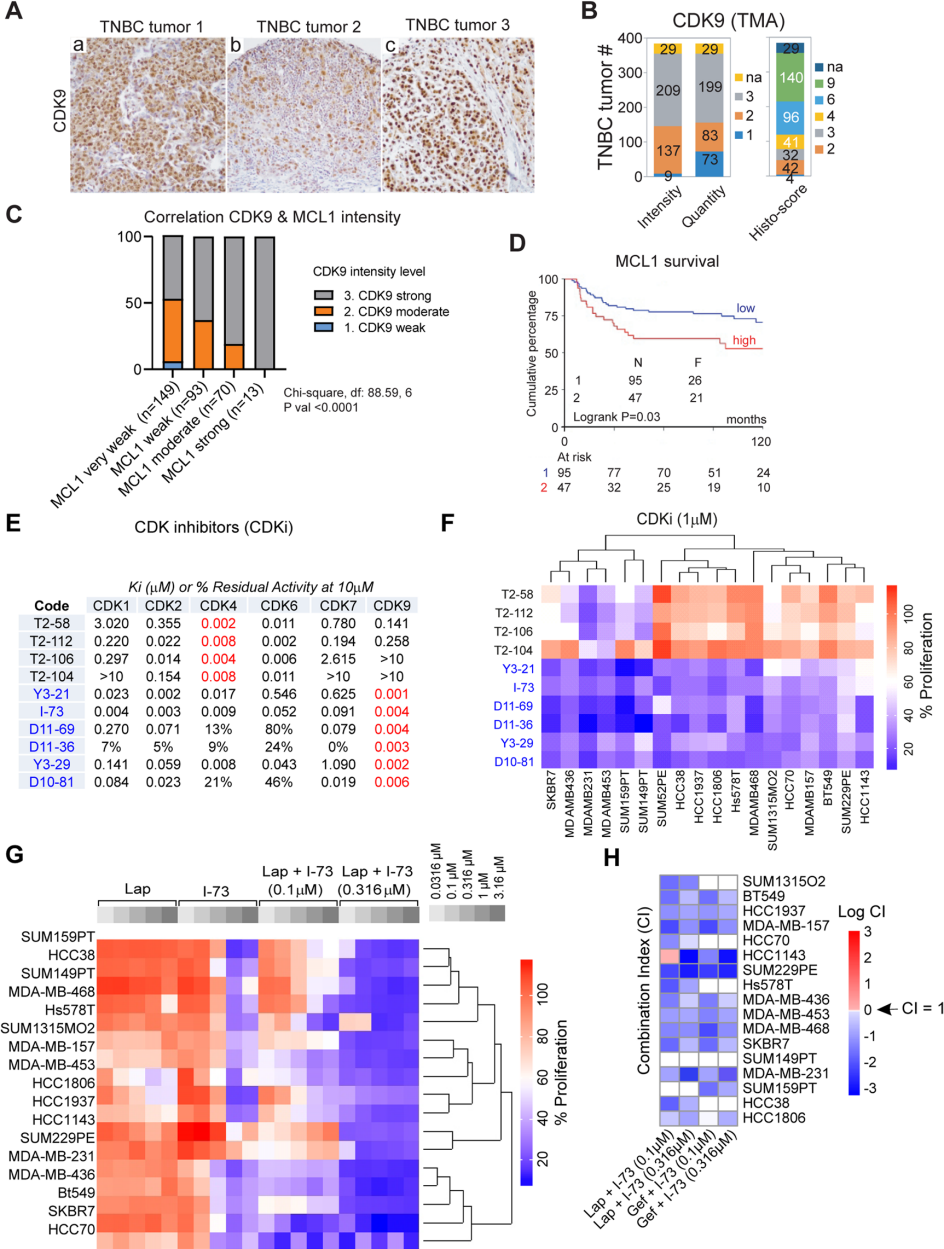
CDK9 is a promising target in triple-negative breast cancer (TNBC) and its inhibition synergizes with EGFR inhibition. (**A**) Representative examples of CDK9 tissue microarray (TMA) staining in TNBC tumours. (**B**) Intensity, quantity and histo-scores (see Methods) for CDK9 TMA staining in 384 TNBC cases. (**C**) Association of CDK9 intensity with MCL1 intensity of TMA’s. Associations were tested using a Chi-square test on the different intensity groups. (**D**) Correlation of MCL1 gene expression with metastasis-free survival of 142 lymph-node negative TNBC patients that did not receive adjuvant or neo-adjuvant systemic therapy. (**E**) CDK inhibitory activity of a set of CDK4 and CDK9 inhibitors indicated as either Ki (µM) or % residual effect after treatment with 10 µM of these inhibitors. (**F**) Effects on proliferation of these CDK4 and CDK9 inhibitors (1 µM) on TNBC cell lines. (**G**) Anti-proliferative effects of combining I-73 (0.1 µM and 0.316 µM) with a concentration range of lapatinib in TNBC cell lines. (**H**) Corresponding combination indexes (CI) calculated from these treatments in Hs578T cells. The CI indexes are presented in natural algorithm (Log CI) and indicate antagonism (CI > 1), additivity (CI = 1) or synergy (CI < 1)

To assess CDK9 as a potential target in TNBC, we evaluated CDK9 expression levels on previously established tissue microarrays (TMA) of 384 TNBC patient tumours [31]. This TMA analysis revealed that the TNBC tumours were broadly CDK9-positive, with around 75% of tumours showing 80–100% positivity with moderate to strong staining intensity (i.e. histological score 4–9, Fig. 1A and B). Previous research has shown that the pro-survival protein MCL1 is quickly downregulated after CDK9 inhibition in mouse models and cell lines, and therefore MCL1 is considered a critical target of CDK9 [23–27]. For that reason, we also evaluated MCL1 levels on TMAs from the same patients. MCL1 levels varied between patient tumour tissues and MCL1 intensity correlated with CDK9 intensity (Fig. 1C). Importantly, high *MCL1* RNA levels in tumour tissue were associated with poorer survival in these patients (Fig. 1D). These data further support the rationale for targeting CDK9 in TNBC.

### TNBC cells are susceptible to CDK9 inhibitors, which can be further potentiated by EGFR inhibitors

To explore the sensitivity of TNBC cells to CDK inhibitors, particularly those targeting CDK9 kinase activity, we treated a panel of 18 TNBC cell lines, representing various TNBC subtypes, with a set of CDK inhibitors [32, 33]. This panel includes highly potent CDK9 inhibitors (Y3-21, I-73, D11-69, D11-36, Y3-29, and D10-81), and CDK4/6 inhibitors (T2-58, T2-112, T-106, and T2-104) (Fig. 1E). Proliferation assays showed that TNBC cell lines were less responsive to CDK4/6 inhibitors but sensitive to CDK9 inhibitors in a concentration-dependent manner (Fig. 1F and Fig. S1A).

For comparative purposes, we evaluated CDK inhibitors currently in clinical trials or approved for clinical use (Fig. S1B). The response to clinically approved CDK4/6-specific inhibitors, such as palbociclib and abemaciclib, mirrored the response to the CDK4/6 inhibitors T2-58, T2-112, T2-106, and T2-104 (Fig. 1F, Fig. S1B). This is consistent with recent reports suggesting that the luminal androgen receptor (LAR) subtype of TNBC is sensitive to CDK4/6 inhibitors like palbociclib and ribociclib [34], the LAR subtype cell line MDA-MB-453 was notably sensitive to CDK4/6 inhibitors. Likewise, the mesenchymal stem-like (MSL) subtype cell line MDA-MB-231 also exhibited high sensitivity to CDK4/6-inhibition [35, 36]. In contrast, TNBC cells were generally susceptible to pan-CDK inhibitors dinaciclib and flavopiridol, but not to roscovitine or P276-00 (Fig. S1B). Of relevance, all 18 TNBC cell lines were sensitive to CDK7 inhibitor THZ1 (Fig. S1B), consistent with previous reports indicating CDK7 as a specific vulnerability in TNBC [37]. This sensitivity pattern closely resembled that observed with CDK9 inhibitors Y3-21, I-73, D11-69, D11-36, Y3-29, and D10-81 (Fig. 1F, Fig. S1A), reinforcing the critical role of CDK9-mediated transcription in TNBC cells.

We previously observed that the CDK9/Cdc7 inhibitor PHA-767491 synergizes with EGFR inhibitors in TNBC cells [29], and that this synergy can be attributed to the inhibition of ABCG2-mediated CDK-inhibitor transport (including I-73) by lapatinib [30]. Building on this, we further evaluated the synergistic potential of two selective CDK9 inhibitors, I-73 and Y3-21, in combination with EGFR inhibitors, lapatinib and gefitinib, across a panel of 18 TNBC cell lines. As expected, co-treatment with I-73 (0.1 µM or 0.316 µM) or Y3-21 (0.0316 µM or 0.1 µM) and lapatinib (0.316–3.16 µM) or gefitinib (0.316–3.16 µM) led to a synergistic reduction in cell proliferation, particularly in EGFR-resistant TNBC cell lines (Fig. 1G and H, Fig. S1C and D). This underscores the ongoing importance of combining CDK9 and EGFR inhibitors to enhance therapeutic efficacy in TNBC.

### CDK9 inhibitors suppress P-TEFb signalling and pro-survival protein expression while simultaneously inducing apoptosis in TNBC cells

**Fig. 2:**
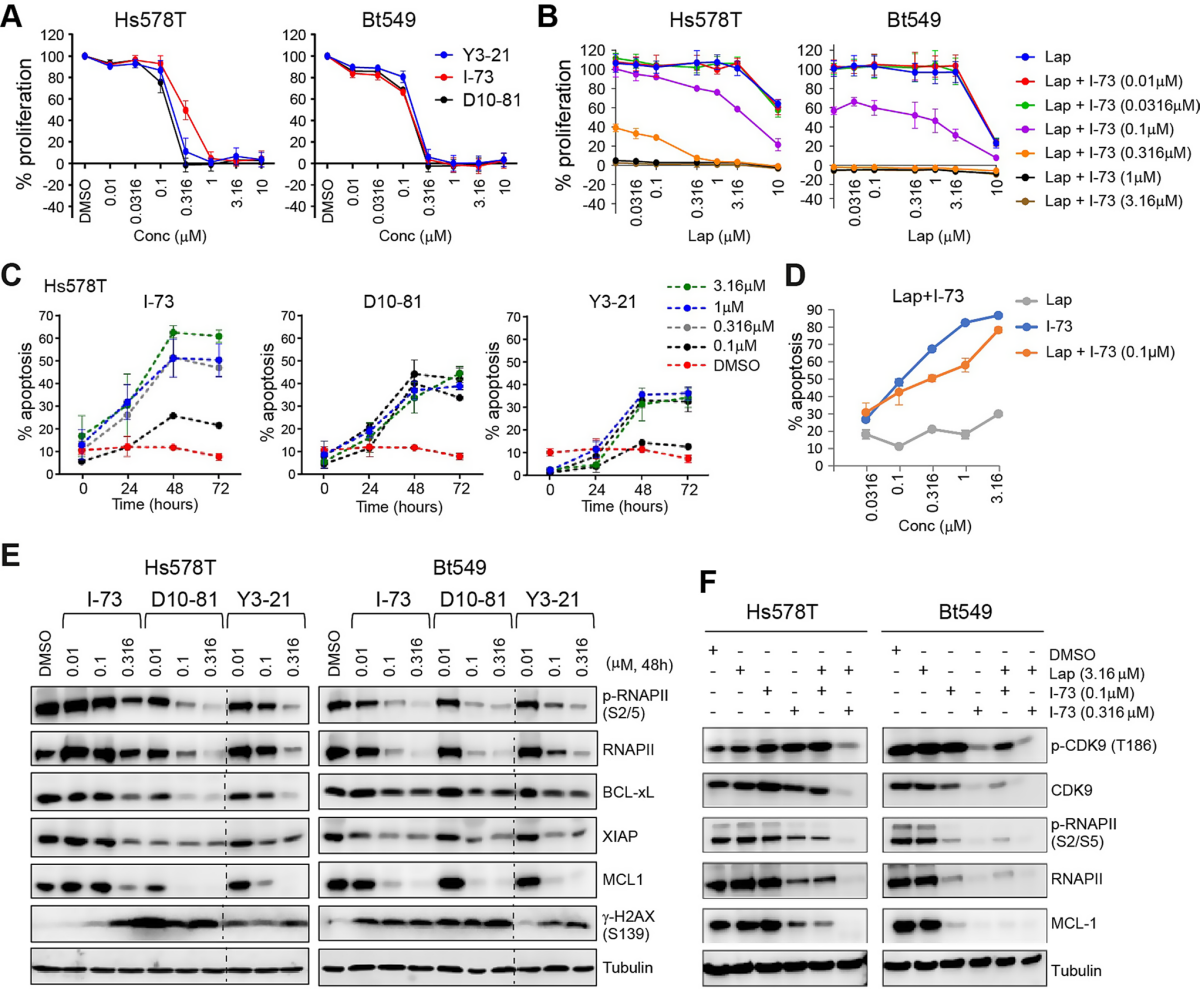
CDK9 inhibitors and combination treatment with lapatinib induce apoptosis and inhibit CDK9 downstream targets. (**A**) Dose-response curves of Y3-21, I-73 and D10-81 in Hs578T and Bt549 cells. (**B**) Dose-response curves of lapatinib with a combination treatment with different doses of I-73 (0.01–3.16 µM). (**C**) Induction of apoptosis (% of annexin V positive cells) after treatment with different doses of I-73, D10-81, Y3-21 (0.1, 0.316 and 1 and 3.16 µM) for 72 h in Hs578T cells. (**D**) Induction of apoptosis after treatment of different doses of lapatinib, I-73, or a combination thereof in Hs578T cells. (**E**) Effect of 48 h of treatment with different doses of I-73, D10-81, Y3-21 (0.01, 0.1 and 0.316 µM) on RNA polymerase II phosphorylation levels, expression of pro-survival proteins BCL-xL, XIAP and MCL1, and H2AX phosphorylation (S139) in Hs578T and BT549 cells. Dotted lines are cropmarks for skipped lanes within the same blot, uncropped blots are in Supplementary File 2. (**F**) Effect of 48-hour combination treatment of lapatinib (3.16 µM) and I-73 (0.1 and 0.316 µM) on CDK9 and RNA polymerase II phosphorylation and MCL1 expression

The anti-proliferative effects of the CDK9 inhibitors, I-73, Y3-21, and D10-81 (Fig. S2A) were subsequently validated in selected TNBC lines Hs578T, BT549 (Fig. 2A and B) and SKBR7 (Fig. S2B and S2C). These CDK9 inhibitors additionally induced apoptosis in TNBC cell lines in a concentration- and time-dependent manner (Fig. 2C, Fig. S2D). While I-73 (at 0.1 µM) and lapatinib (up to 3.16 µM) individually had little effect on apoptosis, their combination resulted in a pronounced increase in apoptosis (Fig. 2D).

Given that the CDK9 inhibitors potently induced cell death, we further evaluated their effect on expression of anti-apoptotic factors in TNBC cells. After 48 h of treatment with D10-81 (0.1 µM), I-73 (0.316 µM) and Y3-21 (0.1 µM), we observed a substantial reduction in total or phosphorylated RNAPII (p-RNAPII) levels, confirming on-target activity at those concentrations in Hs578T, BT549 and SKBR7 cells (Fig. 2E, Fig. S2E). Moreover, these treatments caused marked depletion of pro-survival and anti-apoptotic BCL-2 family members MCL1 and BCL-xL, as well as the X-linked inhibitor of apoptosis (XIAP). The combination of I-73 (0.1 µM) and lapatinib (3.16 µM) further synergistically reduced p-RNAPII, total RNAPII, and MCL1 levels (Fig. 2F, Fig. S2F). Intriguingly, the CDK9 inhibitors also robustly increased H2AX phosphorylation at S139 (γ-H2AX), indicating DNA replication stress and DNA double-stranded breaks in TNBC cells (Fig. 2E, Fig. S2E). To elucidate the dynamics of CDK9 inhibition, we examined changes in protein levels of MCL1, γ-H2AX and p-RNAPII over a 48-hours period post-treatment with D10-81, I-73, and Y3-21 (Fig. S3A-C). p-RNAPII levels reduced within 4 h of treatment, while total RNAPII levels declined after 24 h. In addition, treatment with the CDK9 inhibitors caused rapid downregulation of MCL1, consistent with its short half-life, and induction of H2AX phosphorylation at later timepoints.

### CDK9 inhibitors induce transcriptional reprogramming of multiple oncogenic signalling pathways in TNBC cells

**Fig. 3:**
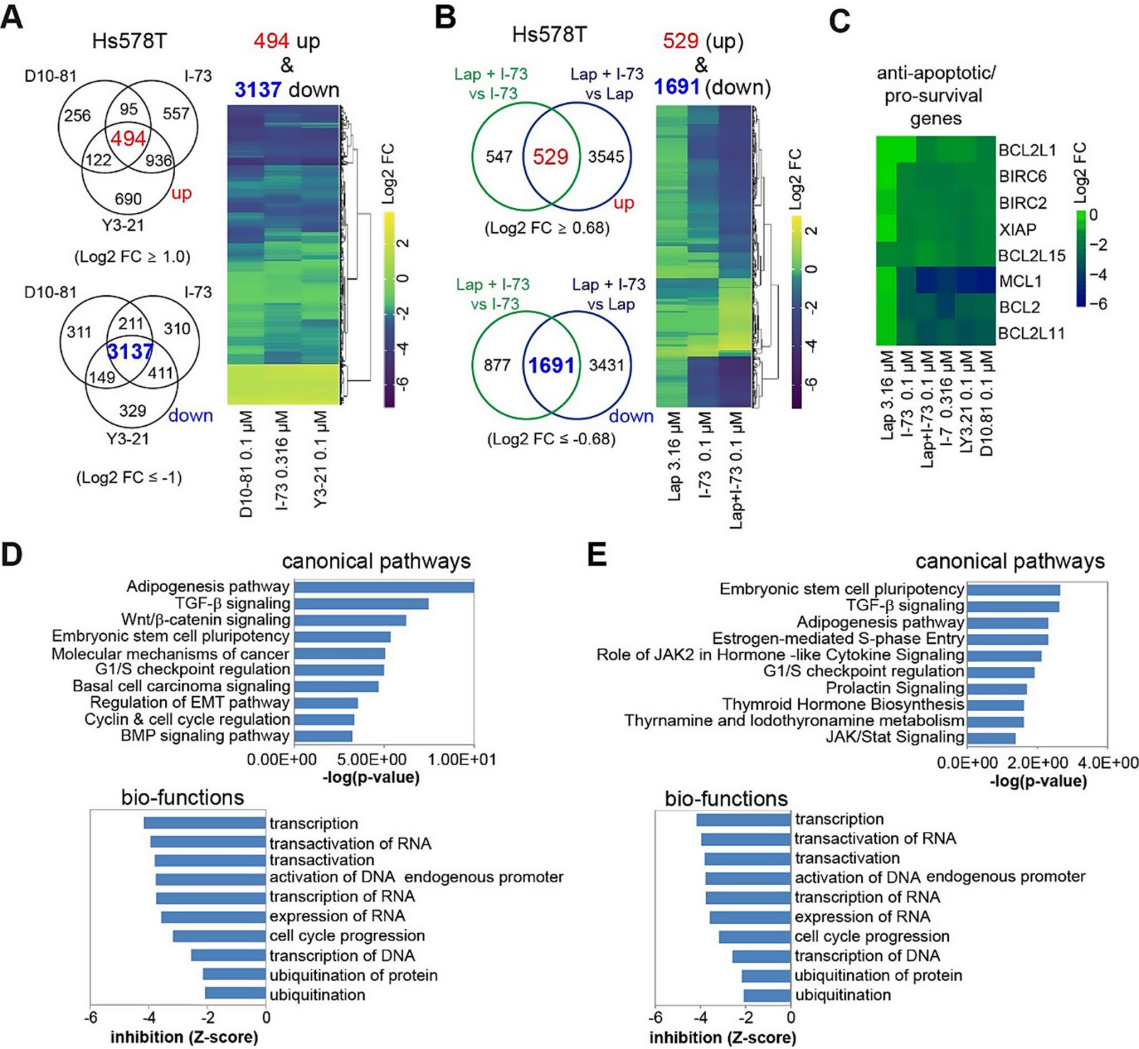
CDK9 inhibitors induce transcriptome reprogramming, which is potentiated by co-treatment with lapatinib in Hs578T cells. (**A**) Overlap between differentially up- (log2 foldchange (Log2FC) > 1) and down-regulated (log2FC<-1) genes after 6 h treatment with D10-81 (0.1 µM), I-73 (0.316 µM), and Y3-21 (0.1 µM) in Hs578T, as determined by RNA-sequencing, and corresponding log2FC of these overlapping genes. (**B**) Overlap between differentially up- (log2FC > 0.68) and downregulated (log2FC<-0.68) genes of synergistic lapatinib (3.16 µM) and I-73 (0.1 µM) treatment, compared to either I-73 (0.1 µM) or lapatinib (3.16 µM) genes, and corresponding log2FC of these overlapping, synergy-related genes. (**C**) Log2FC of anti-apoptotic and pro-survival genes after treatment with these inhibitors compared to DMSO in Hs578T cells. (**D**-**E**) Corresponding ingenuity pathway analysis (IPA) of canonical pathways and bio-functions of the overlapping genes after treatment with CDK9 inhibitors (**D**) or combination treatment of I-73 and lapatinib (**E**)

Since the P-TEFb complex controls transcription, targeting CDK9 would impair the transcriptional program in TNBC. To explore this, we compared the transcriptome of Hs578T cells treated with D10-81 (0.1 µM), Y3-21 (0.1 µM) or I-73 (0.316 µM) for 6 h using RNA-sequencing (Table S1). These inhibitors altered the transcriptional profile of Hs578T cells to a similar extent, indicating a high degree of target specificity and a similar biological mode of action (Fig. 3A). We identified 3631 genes commonly affected by all three CDK9 inhibitors, with 86% (3137 genes) downregulated (Log2 Fold Change (FC) ≤ -1.0) and 14% (494 genes) upregulated (Log2 FC ≥ 1.0) following treatment. Moreover, combining the combination of lapatinib (3.16 µM) with I-73 (0.1 µM) led to substantial downregulation of gene expression, with 2220 genes (Log2 FC ≥ ± 0.68) significantly compared to either mono-treatment. Of those, 1691 genes were downregulated (Fig. 3B). These 2220 genes were further analysed as synergy-related genes in the context of lapatinib and I-73. Most of the 2220 genes were slightly affected by I-73 (0.1 µM) alone, and the addition of lapatinib predominantly enhanced these effects (Fig. 3B). Notably, several pro-survival and anti-apoptotic genes, including *BCL2*, *BCL2L11*, *BCL2L15*, *MCL1* and *XIAP*, were significantly downregulated following treatment with CDK9 inhibitors and the combination of I-73 and lapatinib (Fig. 3C).

**Fig. 4:**
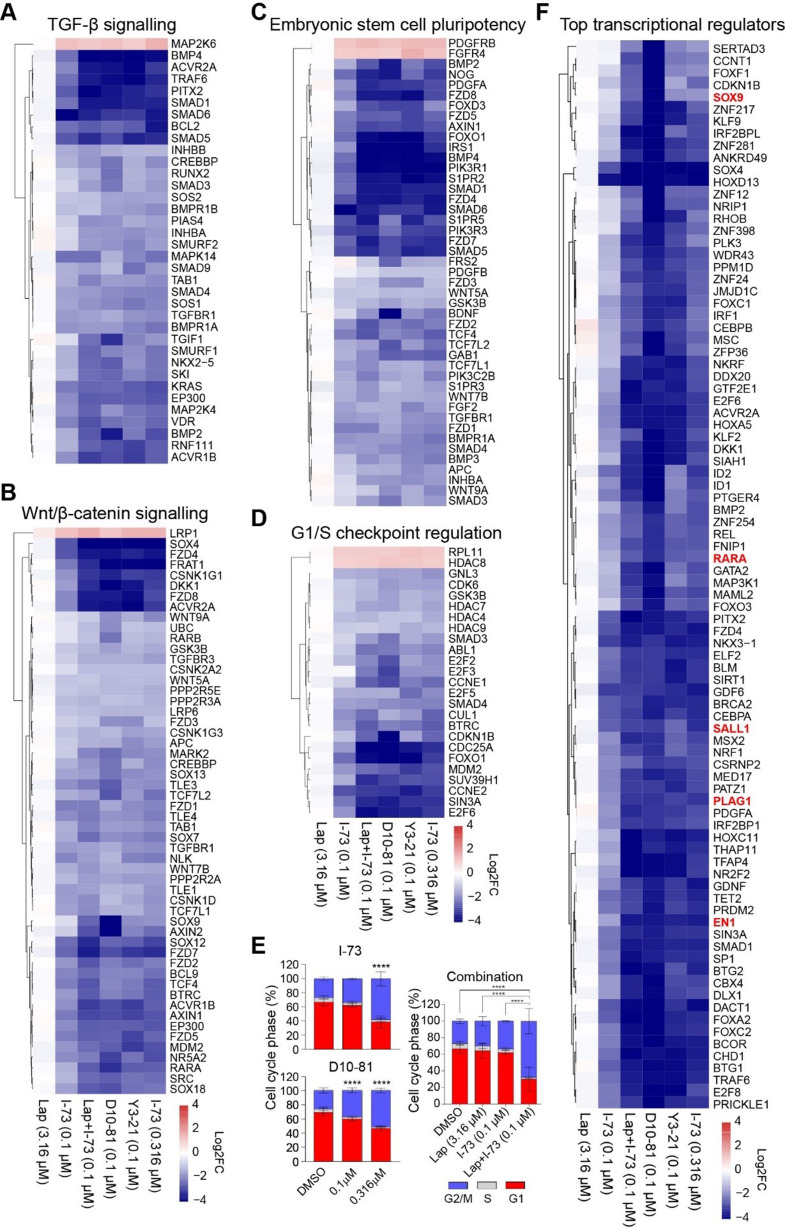
CDK9 inhibitors impair gene expression of critical cancer-related pathways. (**A-D**) Log2FC of differentially expressed genes from TGF-β signaling (**A**) Wnt/β-catenin (**B**), embryonic stem cell pluripotency (**C**), or G1/S checkpoint regulation (**D**) pathways, 6 h after treatment with CDK9 inhibitors and/or combination treatment with lapatinib in Hs578T cells. (**E**) Cell cycle distribution 48 h after treatment with I-73 and D10-81 (0.1 and 0.316 µM) or combination treatment with lapatinib (3.16 µM) and I-73 (0.1 µM) in Hs578T cells. Mean ± standard deviation (*n* = 3). Two-way ANOVA with Tukey’s multiple comparison test; **** *p* < 0.0001. (**F**) Log2FC of top 80 genes (sorted on Log2FC and selected based on Log2FC of CDK9 inhibitors <-1, and for D10-81 <-3), functioning as regulator of mRNA transcription 6 h after treatment with CDK9 inhibitors in Hs578T cells

Ingenuity Pathway Analysis (IPA) further elucidated the canonical pathways and biological functions enriched among the differentially expressed genes (Fig. 3D and E). Analysis of the 2220 synergy-associated genes revealed enrichment in pathways similar to those affected by higher doses of I-73 or other CDK9 inhibitors. These findings reinforce that the combination treatment primarily amplified the effects of I-73. Genes commonly downregulated by these treatments were notably enriched for various oncogenic pathways (Fig. S4), including TGF-β signalling (e.g., *SMAD1*, *SMAD5* and *SMAD6*; Fig. 4A), Wnt/β-catenin signalling (e.g., *SOX4*,* SOX9*,* RARA*,* WNT9A;* Fig. 4B) and embryonic stem cell pluripotency (e.g., *NOG*,* BDNF;* Fig. 4C). Moreover, genes involved in G1/S checkpoint regulation (e.g., *CCNE2*, *E2F2*, *E2F6*) were also strongly downregulated (Fig. 4D). Given these effects on cell cycle-related gene expression, we evaluated their impact on cell cycle progression through FACS analysis. Treatment for 48 h with the (combination) therapies increased cells in the G2/M phase and a reduction of the number of cells in the G1 and S phase (Fig. 4E). This shift aligns with the observed reduction in G1/S-related gene expression (Fig. 4D).

### Transcriptional CDK inhibitors downregulate the expression of transcription factors essential for the proliferation of TNBC cells

**Fig. 5:**
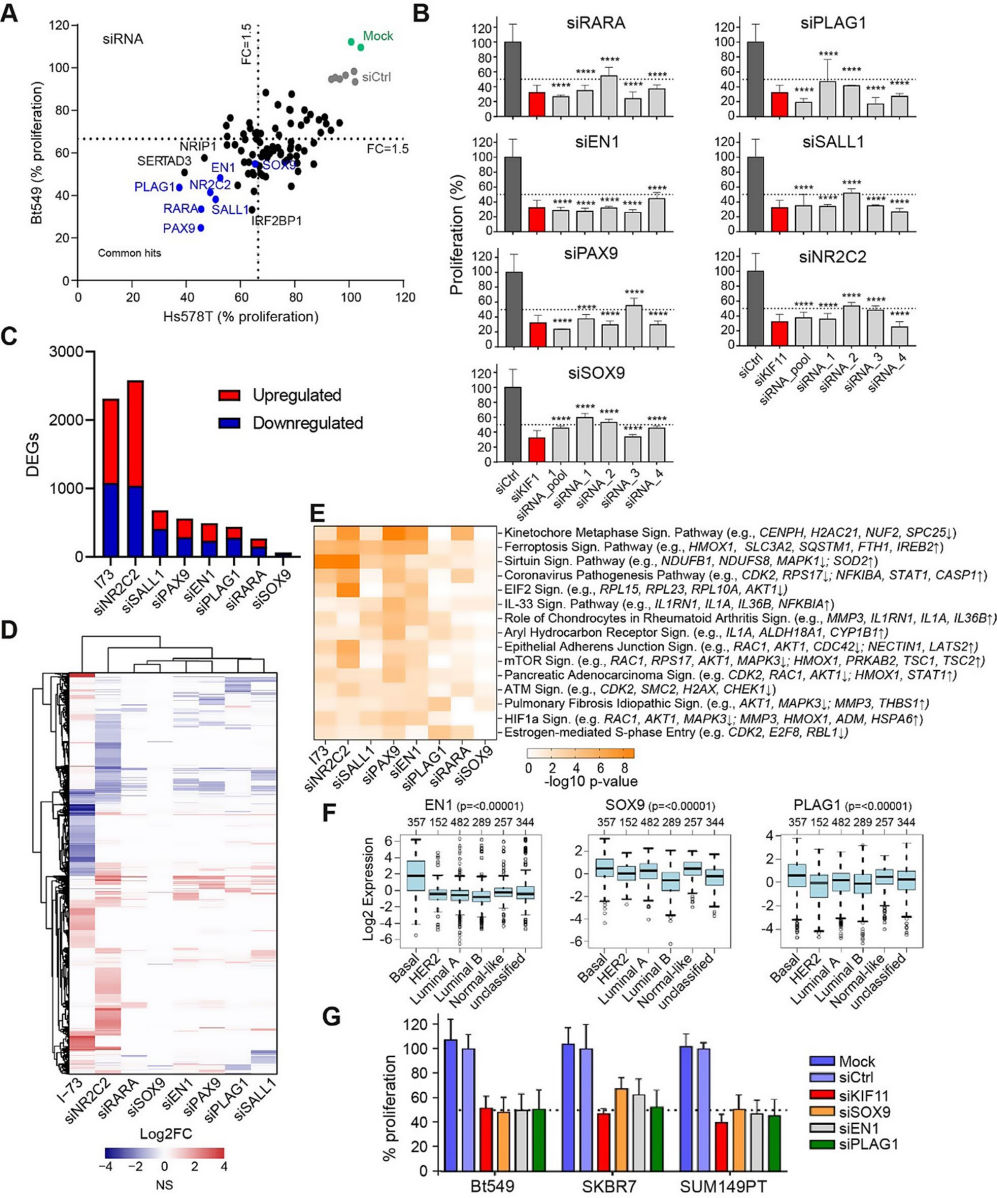
Transcriptional regulators downregulated by CDK9 inhibitors essential for TNBC proliferation. (**A**) Proliferation of Hs578T and BT549 cells after siRNA knockdown of top 80 transcriptional regulator genes. (**B**) SiRNA knockdown of RARA, PLAG1, EN1, SALL1, PAX9, NR2C2, SOX9 using pooled and four individual siRNA sequences. Data shown as mean ± standard deviation (*n* = 2, ****=*P* < 0.0001). One-way ANOVA with Tukey’s multiple comparisons test. (**C**) Number of differentially expressed genes 72 h after knockdown of these genes, and 6 h after I-73 treatment, in Hs578T cells, as determined by TempOseq targeted RNA-sequencing. (**D**) Log2FC of significantly differentially expressed (adjusted p-value < 0.05, log2FC≤-0.5 or ≥ 0.5) genes after these treatments. (**E**) p-values (-log10) of top 15 canonical pathways (sorted on average p-value) enriched among differentially expressed genes after knockdown. Example of genes contributing to enrichment of the pathway in at least two knockdown conditions are indicated. (**F**) Expression of the transcription factors EN1, SOX9 and PLAG1 in the basal-like subtype, across breast cancer intrinsic subtypes (data derived from GOBO). (**G**) Effect of SOX9, EN1 or PLAG1 silencing on the proliferation of BT549, SKBR7 and SUM149PT cells

The biological functions of the genes downregulated by CDK9 inhibitors and the combination treatment with lapatinib were strongly enriched for functions related to RNA transcription regulation. Specifically, among the 564 shared differentially expressed genes associated with transcriptional regulation, 543 were downregulated by the CDK9 inhibitors (Table S1). This downregulation likely impacts gene expression in TNBC cells significantly. To determine if the downregulation of these transcriptional regulators could contribute to the antiproliferative effects of CDK9 inhibitors, we focused on the 80 most strongly affected transcription factor genes (Log2 FC ≤ -3.0 for D11-81, Fig. 4F) and silenced them using siRNA in Hs578T and BT549 cells (Fig. 5A). Silencing several of these transcriptional regulators, including *PLAG1*, *SOX9*, *SERTAD3*, *RARA*, *PAX9*, *NR2C2*, *SALL1* and *EN1*, resulted in a strong reduction in cell proliferation by at least 50% compared to control in both cell lines (Fig. 5A, Fig. S5A and S5B). Further validation through deconvolution screening with pooled and single siRNAs confirmed that knockdown of *RARA*, *EN1*, *PAX9*, *SOX9*, *PLAG1*, *SALL1* and *NR2C2* - using at least 2 out of 4 single siRNAs - reduced cell proliferation by over 50% in Hs578T (Fig. 5B) and BT549 cells (Fig. S5C). Overall, these data demonstrate that CDK9 inhibition suppresses the expression of transcription factors that are essential for TNBC cell proliferation.

To further understand the role of these seven transcription factors in transcriptional regulation, we examined their impact on gene expression and how their downregulation contributes to the overall transcriptional changes induced by CDK9 inhibition. We performed whole genome targeted RNA sequencing three days after knockdown of *RARA*, *EN1*, *PAX9*, *SOX9*, *PLAG1*, *SALL1* and *NR2C2*, or after 6 h of inhibition with I-73 (0.316 µM) in Hs578T cells (Table S1). The knockdown of most of these genes had a much more selective effect on gene expression compared to I-73 treatment (1083 downregulated, 1230 upregulated), except for knockdown of NR2C2 which strongly impacted gene expression (1041 downregulated, 1439 upregulated) (Fig. 5C). The transcriptomic profiles resulting from the knockdown of *RARA*, *PLAG1*, *EN1*, *SALL1*, *PAX9*, and *NR2C2* differed substantially from those induced by I-73 treatment (Fig. 5D), suggesting that downregulation of these transcription factors is not the primary driver of the broader gene expression changes observed following I-73 exposure. However, IPA of the differentially expressed genes following knockdown of these transcriptional factors suggested that *NR2C2*, *PAX9* and *EN1* regulate various cell cycle-related genes, such as those involved in kinetochore metaphase signalling, EIF2 signalling and G1/S checkpoint regulation. They also influence DNA damage response pathways, including ATM signalling, which are similarly affected by I-73 (Fig. 5E, Fig. S4D). Altogether, these results suggest that while the transcription factors downregulated by CDK9 inhibitors might contribute to some of the downstream phenotypic effects of these inhibitors, they do not account for the overall transcriptional effects induced by the CDK9 inhibitors.

To further determine the clinical relevance of the downregulated transcription factors concerning TNBC proliferation, we analysed their expression in a cohort of 1881 breast tumours, stratified by PAM50 subtypes [38], using the Gene Expression-Based Outcome for Breast Cancer Online (GOBO) dataset [39]. We observed that the transcription factors *EN1*, *SOX9* and *PLAG1* were significantly higher expressed in basal-like breast tumours (which include approximately 80% TNBC [40]), compared to the other breast tumour subtypes (Fig. 5F). To further validate their role in TNBC proliferation, we silenced these three putative TNBC-specific transcription factors in two additional TNBC cell lines, SKBR7 and SUM149PT. This knockdown significantly reduced cell proliferation in both cell lines (Fig. 5G), further underscoring the essential roles of these transcription factors in driving the proliferation of TNBC cells.

### I-73 and lapatinib synergistically inhibit tumour growth in vivo

**Fig. 6:**
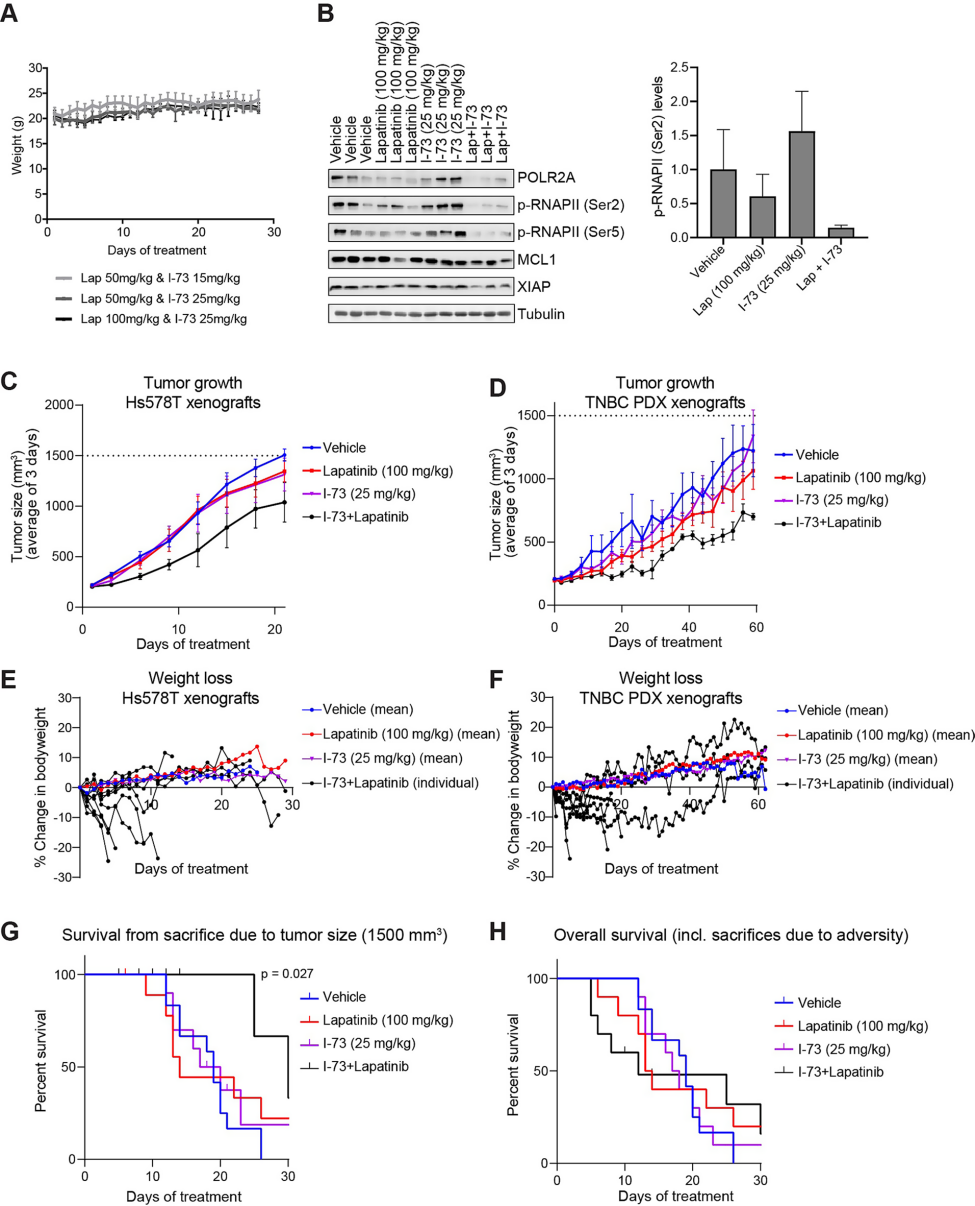
Combination treatment of CDK9 inhibitor I-73 and lapatinib synergizes to inhibit tumour growth, but also induces toxicity, in Hs578T and patient-derived xenografts mouse models. (**A**) Weight of non-tumour bearing mice after treatment with different doses of lapatinib (50 and 100 mg/kg) and I-73 (15 mg/kg and 25 mg/kg) (*n* = 4). (**B**) Effect on RNA polymerase II phosphorylation and MCL1 expression in tumour cells after 5 days treatment with I-73 (25 mg/kg) and/or lapatinib (50 mg/kg) of Hs578T xenografted mice (*n* = 3). (**C**-**D**) Tumour growth over time after treatment with lapatinib, I-73 or the combination thereof in Hs578T (**C**) or patient-derived xenograft (**D**) mouse models. Data are the mean (± SEM) of all mice (*n* = 11,11, 8 and 8 per treatment group for Hs578T xenografts, and *n* = 5, 6, 7 or 8 per treatment group for PDX xenografts for vehicle, lapatinib monotherapy, I-73 monotherapy or lapatinib and I-73 combination therapy, respectively). (**E**-**F**) Percentage of change in body weight after treatment with vehicle, lapatinib or I-73, or the combination thereof compared to the start of treatment in Hs578T xenograft (**E**) or PDX (**F**) mice. Shown data are the mean (± SEM) of all mice for each of the treatment groups, except the I-73 and lapatinib combination (each individual mouse shown). (**G**) Percentage of mice surviving without sacrifice due to tumour size of 1500 mm^3^ after these treatments in Hs578T xenograft models, excluding mice sacrificed due to loss of body weight or showing severe discomfort. (**H**) Percentage of overall survival (i.e. surviving fraction without sacrifice for tumour size, weight loss of mice, or spontaneous deaths) after these treatments in Hs578T xenograft models

Previous studies have established the safety and efficacy of I-73 in various mouse xenografts, but never in a mouse model for TNBC [41–43]. To assess its efficacy against TNBC, we investigated both single-agent and combination treatment of I-73 and lapatinib on TNBC in vivo. We aimed to determine the maximum tolerated dose by treating three groups of non-tumour-bearing mice daily (QD) for 28 days by oral administration (PO) with the following regimens: I-73 (15 mg/kg) plus lapatinib (50 mg/kg), I-73 (25 mg/kg) plus lapatinib (50 mg/kg), or I-73 (25 mg/kg) plus lapatinib (100 mg/kg). None of these treatment regimens caused a reduction in body weight or induced any other signs of discomfort in the mice, indicating that these doses were well-tolerated (Fig. 6A). We further evaluated the impact of the combination treatment on P-TEFb downstream targets in Hs578T xenografted tumours. Five days of treatment (QD, PO) with I-73 (25 mg/kg) plus lapatinib (100 mg/kg) synergistically reduced the levels of p-RNAPII, confirming the pharmacological activity of the drugs (Fig. 6B). Additionally, the combination treatment slightly reduced MCL1 levels.

We then evaluated the impact of this I-73 and lapatinib combination regimen on tumour growth in Hs578T xenograft and TNBC patient-derived xenograft (PDX) mouse models. This combination treatment (25 mg/kg I-73, 100 mg/kg lapatinib, QD, PO) effectively inhibited tumour growth in both Hs578T xenograft (up to 30 days treatment) and PDX mouse models (up to 65 days treatment) (Fig. 6C and D). However, it also resulted in body weight loss in 4 out of 8 mice (Fig. 6E and F). Despite a significant reduction in tumour-related deaths (sacrifice at 1500 mm³) in the Hs578T xenografts with the combination treatment, overall survival did not improve due to the adverse effects associated with the therapy (Fig. 6G and H). While these results demonstrate the in vivo pharmacological synergistic efficacy of I-73 and lapatinib, further investigation is warranted to understand the mechanism of toxicity and to develop optimal dosing strategies, including co-treatment and sequential treatment regimens.

## Discussion

Inhibiting transcriptional CDKs has emerged as an attractive therapeutic strategy to exploit the dependence of various cancers on aberrant transcriptional programs. Our study demonstrates that CDK9 is an effective therapeutic target against TNBC, an aggressive subtype of breast cancer for which currently limited treatment options are available [2]. The CDK9 inhibitors tested here, including I-73, D10-81, Y3-21, D11-36, D11-69, and Y3-29, were highly potent anti-proliferative agents against TNBC cells. Moreover, we show that the effect of I-73 and Y3-21 can be effectively augmented with EGFR inhibitors.

While CDK9 itself is not known to be strongly deregulated in TNBC, our results show that its targeting may impact several pathways and processes that are deregulated. Cancer cells are dependent on equilibrium regarding pro- and anti-apoptotic factors for their survival, high proliferative capacity and to become resistant to external stresses such as anti-cancer drugs [44–46]. Therapies targeting these proteins, such as BH3 mimetics, have therefore gained substantial interest [47]. However, resistance against these therapies is frequently induced due to the upregulation of other pro-survival proteins, such as MCL1 upregulation after Bcl-2 inhibition [48]. Here, we demonstrate that the CDK9 inhibitors D10-81, Y3-21 and I-73 potently induced apoptosis in different TNBC cell lines, accompanied by reduced expression of multiple anti-apoptotic and pro-survival factors. The simultaneous downregulation of multiple (anti-apoptotic) genes may therefore be an effective strategy to prevent drug resistance. In addition, we show that these CDK9 inhibitors reduced gene expression associated with differentiation state and stemness, which might affect cancer stem cell population, cell differentiation and/or cancer cell plasticity (e.g. proliferation/dormancy and epithelial-to-mesenchymal plasticity). Indeed, these alterations in gene expression might explain the results from a previous study demonstrating that CDK9 inhibition disrupted breast cancer stem cells [49, 50].

Moreover, we observed a strong enrichment of downregulation of many transcription factors, which may have detrimental secondary effects. Similarly, transcription factors whose expression is regulated by super-enhancers and that drive oncogenic transcriptional programs were previously shown to be sensitive to inhibition of CDK7 and BRD4 [16, 37, 51–53]. Our analysis of transcription factors whose expression was inhibited by D10-81, I-73 and Y3-21, revealed a group of transcription factors, including *RARA*, *EN1*, *SOX9*, *PLAG1*, *SALL1*, *PAX9* and *NR2C2.* Silencing of these transcription factors strongly inhibited TNBC cell proliferation and further impacted gene expression, especially for *NR2C2*. NR2C2 is a relatively poorly studied orphan nuclear receptor, which can act both as transcriptional activator as well as repressor, and of which the endogenous ligand is still unknown. The strong impact on TNBC transcriptome and proliferation thus warrants its further investigation. In addition, *EN1*, *SOX9* and *PLAG1* genes were significantly higher expressed in basal-like breast tumours. *EN1* and *SOX9* have previously been identified as super-enhancer driven transcription factors in TNBC cells and their expression is also highly sensitive to CDK7 inhibitor THZ1 [37]. Despite their functional and clinical relevance, knockdown of these transcription factors showed limited overlap with the I-73 transcriptomic profile, suggesting their downregulation is not central to the broader transcriptional effects of the CDK9 inhibition. Nevertheless, CDK9 inhibitors may be used to indirectly target TNBC driven by certain of these transcription factors.

Although our in vivo investigations confirm the potential synergy between CDK9 and EGFR inhibitors, further exploration of their safety and underlying mechanisms of action is essential. The combined treatment of I-73 and lapatinib exhibited no adverse effects in non-tumour bearing mice; however, it unexpectedly negatively impacted mice bearing tumours. The presence of a tumour could have influenced the overall health status of these mice, rendering them more susceptible to potential negative effects from the combination treatment, in contrast to healthy mice. Alternatively, the observed side effects could stem from tumour-related events, such as tumour lysis syndrome, a phenomenon previously also associated with pan-CDK inhibitors dinaciclib and flavopiridol in clinical trials [54, 55]. Interestingly, such effects have not been reported in the initial phase I study of the more selective CDK9 inhibitor, BAY1251152 (VIP152), suggesting an enhanced safety profile for more selective CDK9 inhibitors [56]. As monotherapy, I-73 (dosages up to 100 mg/kg) exhibits minimal toxicity in mice and against a variety of non-transformed human cells [41–43, 57], suggesting a potential safety issue associated with its use in combination with lapatinib. We have recently demonstrated that this observed synergistic interaction is caused by inhibition of ABCG2 by several kinase inhibitors, including lapatinib [30]. This inhibition potentially leads to accumulation of the drug in organs expressing high levels of ABCG2, such as the liver. Therefore, developing CDK9 inhibitors that are not an ABCG2 substrate may be more beneficial than inhibition of ABCG2 through lapatinib. Alternatively, lower concentrations of lapatinib might be sufficient to inhibit ABCG2 activity in cancer cells, but not in organs expressing higher levels of ABCG2 (e.g. liver). Therefore, lower concentrations of lapatinib could facilitate between discrimination of the tumour compared to healthy organs and limit toxicity, while not limiting efficacy.

The mechanism underlying the toxicity observed with combined EGFR/CDK9 inhibition remains unclear and may reflect either on-target effects of dual pathway suppression or off-target activities of I-73 (e.g., CDK1/2 inhibition). Although the different CDK9 inhibitors tested here produced highly similar outcomes in vitro, off-target activities cannot be fully excluded and may contribute to the observed phenotypes in vitro and in vivo. In addition, our transcriptomic analyses was restricted to one TNBC cell line, and extension to additional cell lines will be necessary to assess the generalizability of our transcriptomics findings.

Overall, our study underscores the potential of targeting CDK9 to disrupt TNBC transcriptional programs and demonstrates the efficacy of a new panel of potent CDK9 inhibitors. However, it also highlights the need for careful assessment of potential toxicity when using CDK9 inhibitors in combination with tyrosine kinase inhibitors, particularly those that may rely on ABCG2 as a mechanism of synergy. This research reveals that the relationship between target specificity, anti-TNBC effects, and adverse effects requires further investigation. Such studies will be crucial for the clinical development of CDK9 inhibitors, ensuring their safety and effectiveness in treating TNBC.

## Materials & methods

### Inhibitors and antibodies

CDK4/6 inhibitors T2-58, T2-104, T2-106 and T2-112, and CDK9 inhibitors I-73 (also named CDKI-73 [57]), D10-81, D11-36, D11-69, Y3-21, and Y3-29 were synthesised as reported previously [58]. Inhibition of CDK activity by these inhibitors was measured by radiometric assay, as described previously [32]. Pan-CDK inhibitors dinaciclib (S2768), flavopiridol (S1230), P276-00 (S8058) and roscovitine (S1153), CDK7 inhibitor THZ1 (S7549), CDK4/6 inhibitors abemaciclib (S7158) and palbociclib (S1579), and EGFR inhibitors lapatinib (S1028) and gefitinib (S1025) were purchased at Selleckchem. All compounds were prepared in DMSO at 10 mM concentration. All primary antibodies used for western blotting were commercially available, including phospho-CDK9 (T186; CST #2549), CDK9 (CST #2316), phospho-RNAPII Ser2/Ser5 (CST #4735), phospho-RNAPII Ser2 (CST #13499), phospho-RNAPII Ser5 (CST #13523), RNAPII (CST #2629), Survivin (CST #2808), BCL-xL (CST #2764), XIAP (CST #2045), MCL1 (CST #5453), γ-H2AX (S139; CST #2577) and tubulin (Sigma Aldrich T-9026).

### Cell culture and compound screening

Authenticated TNBC cell lines used were representative for different TNBC subtypes [1], including basal-like 1 (BL1) HCC38, HCC1143, HCC1937 and MDA-MB-468, basal-like 2 (BL2) HCC70, HCC1806 and SUM149PT, mesenchymal (M) BT549 and Hs578T, mesenchymal stem-like (MSL) MDA-MB-157, MDA-MB-231, MDA-MB-436 and SUM159PT, luminal androgen receptor (LAR) MDA-MB-453, and unclassified SKBR7, SUM52PE, SUM229PE and SUM1315MO2. All TNBC cell lines were maintained in RPMI-1640 medium (Gibco, ThermoFisher Scientific) supplemented with 10% FBS (ThermoFisher Scientific; 10270106) and 25 IU/ml Penicillin and 25 µg/ml Streptomycin (ThermoFisher Scientific; 15070-063). Cells were cultured in a humidified incubator at 37 °C, 5% CO_2_.

### Sulphorhodamine B cell proliferation assay

96 h post-exposure to inhibitors cells were fixed by adding 30 µl/well of 50% w/v trichloroacetic acid (Sigma; T9159). Plates were incubated at 4 °C for 1 h before being washed five times in H_2_O and dried overnight at room temperature. The following day, cells were stained with 60 µl per well of 0.4% w/v sulphorhodamine B (SRB)/1% v/v acetic acid solution (Sigma; 341738) for 2 h before being washed four times with 1% v/v acetic acid and dried overnight at room temperature. SRB was subsequently dissolved by adding 150 µl/well of 10 mM Tris and plates were placed on a shaker for 2 h at room temperature. Absorbance at 540 nm was then measured using TECAN Infinite ^®^ M1000 (Tecan Trading AG). Data were normalised to DMSO control values, and the percentage of proliferation relative to DMSO and day 0 values, fixed on the day of treatment, was presented.

### Annexin V cell death assay

Cells were seeded at in 96-well µCLEAR plates (Greiner; 655090). Prior to drug treatment, cells were stained with Hoechst 33,258 (200 ng/ml) for 45 min at 37 °C, 5% CO_2_. Subsequently, cells were treated with inhibitors in medium containing AlexaFluor-633-labeled Annexin-V (0.9 µg/ml). At multiple timepoints after treatment, cells were imaged using BD Pathway 855 microscope (BD Biosciences). An image analysis pipeline was created in Cell Profiler involving segmentation based on Hoechst 33,258 staining to identify the primary cell objects followed by secondary Annexin V-positive objects. Annexin V-positive cells were defined based on an intensity threshold. This threshold was set based on the negative (DMSO) and positive (doxorubicin) controls. For each image the sum of all nuclei and the sum of the Annexin V-positive cells was determined followed by calculation of the percentage of Annexin V-positive cells.

### Cell cycle analysis

Cells were seeded in 6-well plates and the following day, cells were treated with drugs for 48 h. Detached cells and attached cells were collected during trypsinisation and collected through centrifugation (1000 rpm, 5 min, 4 °C). Pelleted cells were re-suspended in 200 µl ice-cold 1 mM EDTA in PBS and fixed by adding 800 µl ice-cold 100% ethanol (-20 °C). Cells were washed twice with PBS (followed by centrifugation, 1000 rpm, 5 min, room temperature) and pellet was re-suspended in 250 µl of 3 µM DAPI (Sigma Aldrich, 10236276001) in staining buffer (100 µM Tris pH 7.4, 150 mM NaCl, 1 mM CaCl_2_ and 0.5 mM MgCl_2_). After 15 min cells were filtered through 70 μm EASYstrainer filters followed by FACSCanto II analysis (BD Biosciences). Data were analysed using FlowJo V10.

### Western blotting

Cell lysates were harvested in RIPA lysis buffer with 1:100 protease inhibitor cocktail (Sigma; P8340). Cellular proteins were denatured by addition of sample buffer containing 10% β-mercaptoethanol (Acros Organics 125472500). Equal amounts of proteins were subsequently loaded into polyacrylamide gels (7.5–15% depending on desired resolution), resolved using SDS-PAGE and subsequently transferred to PVDF membranes (Merck Chemicals; IPVH00010) overnight. PVDF membranes were then blocked with 5% bovine serum albumin (Sigma Aldrich A9647) in Tris-buffered saline containing 0.05% Tween20 and incubated overnight with primary antibodies. Subsequently, membranes were incubated with HRP-conjugated or fluorescent secondary antibodies. Signals were visualized on the Amersham Imager 600 (GE Healthcare Life Sciences) by Cy5 fluorescence or chemiluminescence after staining with ECL (Prime) Western Blotting Detection Reagent (GE Healthcare Life Sciences). Uncropped and protein ladder images of blots from Figs. 2 and 6 are in Supplementary File 2.

### RNA sequencing

Hs578T cells seeded into 6-well plates were treated with CDK9 inhibitors D10-81 (0.1 µM), Y3-21 (0.1 µM) or I-73 (0.316 µM). For combination treatments, cells were treated with I-73 (0.1 µM), lapatinib (3.16 µM) and a combination thereof. After 6 h of treatment, extraction of RNA was performed using RNeasy Plus Mini Kit (QIAGEN^®^) and RNA was stored at -80 °C. Transcriptome RNA-Sequencing (RNA-Seq) was performed using Illumina high-throughput RNA sequencing. DNA libraries were prepared from the samples with the TruSeq Stranded mRNA Library Prep Kit. The DNA libraries were sequenced according to the Illumina TruSeq v3 protocol on an Illumina HiSeq2500 sequencer. Paired-end reads of 100 base-pairs in length were generated. Alignment was performed using the STAR aligner (version 2.4.2a) against the human GRCh38 reference genome. Marking duplicates, sorting and indexing were performed using Sambamba (version 0.6.6). Gene counts were quantified using the FeatureCounts HTSeq based on the ENSEMBL gene annotation for GRCH38 (release 84). RNA-Seq data was normalised using DESeq2 and differential gene expression was presented in Log2 fold change (Log2 FC) scales [59]. For the effects of single treatments, genes with significant down- or up-regulation (Log2 FC ≥ ± 1.0) under indicated conditions compared to DMSO were evaluated for pathway enrichment using Ingenuity Pathway Analysis (IPA, QIAGEN^®^). For the effect of combination treatments, genes with a significant down- or up-regulation (Log2 FC ≥ ± 0.68, or FC ≥ ± 1.5) under the combination treatment compared to each single treatment (lapatinib and I-73 separately) were evaluated for pathway enrichment using IPA. A less stringent cut-off was used for the synergistic combination treatment, as the effects of single treatment with I-73 (0.1 µM) were mostly augmented, thus not reaching similarly high fold-changes as when compared to DMSO.

### SiRNA transfection

Cells were seeded in 96-wells plates and transfected with 50 nM siGENOME siRNAs (Dharmacon) per 96-well using INTERFERin transfection reagent (Polyplus). The following day medium was refreshed. 48 h post-transfection, cells were lysed for western blot to confirm knockdown efficiency. 96 h post-transfection cells were fixed for SRB proliferation assay.

### TempO-Seq targeted RNA sequencing

TempO-Seq targeted RNA sequencing was performed after knockdown or I-73 treatment. 72 h after knockdown, or 6 h after I-73 treatment, cells were washed with PBS and lysed using TempO-Seq lysis buffer (BioClavis) for 15 min at room temperature and stored at -80 °C. Whole transcriptome TempO-Seq RNA-sequencing and generation of counts per probe was performed by BioClavis. Counts were normalized and differential gene expression was analysed using DeSeq2 [60]. Genes with significant down- or up-regulation (Log2 FC ≥ |0.5|, padj < 0.05) under indicated conditions were evaluated for pathway enrichment using IPA.

### Primary breast cancer tissues and derived tissue micro-arrays for CDK9 and MCL1 immunohistochemistry

The tissue microarray (TMA) used here has been described previously [31]. Briefly, to construct TMAs a total of 412 primary breast cancer tissues were collected from Erasmus University Medical Center pathology archive, and subsequently reviewed by a trained pathologist. The TMAs were prepared using an ATA27 (Beecher Instruments, Sun Prairie, WI, USA). For every tissue, a specialized breast pathologist marked the tumour area, from which three different cores (diameter: 0.6 mm) were taken as biological replicates and transferred in a TMA recipient block. Tissue cores of 0.6 mm were taken from each tissue paraffin block and transferred in triplicate into a TMA recipient block. Stained TMA slides were digitalized and analysed using Slidepath software (Leica Microsystems, Solms, Germany). Expression of ER, PR and Her2 proteins was determined by IHC staining, and fluorescence in situ hybridization was performed when staining of Her2 was scored as 2+ (i.e. uncertain amplification), to assess possible amplification of this gene. Samples were excluded for further analysis when tissues had unclear expression of ER, PR or Her2, no information on lymph-node status and/or adjuvant systemic chemotherapy, and/or poor quality of CDK9 or MCL1 staining (defined as: heterogeneous staining between cores belonging to the same specimen and/or lack of triplicate staining due to lack of at least one core), resulting in a final number of 384 tissues (for CDK9 staining) and 325 tissues (for MCL-1 staining) for data analysis, respectively. This study was approved by the Medical Ethics Committee of the EMC, The Netherlands (MEC 02.953) and was performed in accordance to the Code of Conduct of the Federation of Medical Scientific Societies in The Netherlands, and wherever possible, we adhered to the Reporting Recommendations for Tumour Marker Prognostic Studies (REMARK) [61].

For immunohistochemistry, sections of 4 μm of the TMAs were incubated for 1 h at room temperature with CDK9 (CST, #2316) or MCL1 antibody (Proteintech, 16225-1-AP). Antigen retrieval for CDK9 was performed prior to antibody incubation by heating the slides for 40 min at 95 °C and washing with Dako antigen retrieval solution (pH = 6; DakoCytomation, Carpinteria, CA, USA) when the slides were cooled down to room temperature. For MCL1, antigen retrieval was performed by heating the slides for 20 min at 95 °C and washing with Dako antigen retrieval solution (pH = 9; DakoCytomation, Carpinteria, CA, USA). Staining was visualized by anti-rabbit EnVision+ ^®^ System-HRP (DAB) (DakoCytomation, Carpinteria, CA, USA). CDK9 and MCL1 staining was separately scored in either both percentage of positive invasive breast carcinoma cells and staining intensity (CDK9) or only in staining intensity (MCL1) by two independent observers. MCL1 quantities were not scored because of low heterogeneity between cells from one sample. For the percentage of CDK9-positive invasive tumour cells, three categories were scored: <90% positive (1), 90–95% positive (2) or 95–100% (3) positive. CDK9 intensity values were scored as weak (1), moderate (2) or strong (3). MCL1 intensity values were scored as either negative/very weak (0), weak (1), moderate (2) or strong (3). All cores present on the three TMAs were scored by an experienced researcher in a blind manner. Staining scores of triplicate cores were validated by a second experienced researcher, who was extensively trained by a specialized breast pathologist. In order to combine score CDK9 intensity and percentage positive as measured by IHC, intensity and quantity categories were transformed into numerical values (as indicated above) and a ‘histo-score’ was calculated with the following formula: *Histo-score* = (*intensity value*) *x* (*quantity value*).

### Clinical evaluation of MCL1 gene expression

The clinical relevance of MCL1 gene expression was evaluated using in-house and publicly available gene expression data of lymph-node negative (LNN) primary breast cancer patients who did not receive adjuvant or neo-adjuvant systemic therapy, as well as available metastasis-free survival data, leading to a cohort of 867 patients, of which 142 were classified as TNBC. Data were gathered from Gene Expression Omnibus (http://www.ncbi.nlm.nih.gov/geo/) entries GSE2034, GSE5327, GSE2990, GSE7390 and GSE11121, with all data available on Affymetrix U133A chip. Raw cel files were processed using fRMA parameters (median polish) after which batch effects were corrected using ComBat [62, 63].

### In vivo Hs578T xenograft and patient-derived xenograft models

A maximum tolerated dose (MTD) study was first performed on female BALB/c nude (cAnN/Rj) mice. Mice were treated daily (*n* = 4 per treatment arm) by oral gavage for 28 days with a combination of lapatinib (50 mg/kg or 100 mg/kg) and I-73 (15 mg/kg or 25 mg/kg). Body weight was measured daily.

For the pharmacodynamics study, 1 million Hs578T cells per mice were xenografted into the 4th mammary gland of female BALB/c nude (cAnN/Rj) mice. Starting from the second week after injection, tumour size was measured once every two days. When the tumours reached the volume of 500–750 mm^3^ the mice were randomly stratified into one of the treatment arms (*n* = 3 per treatment arm), the treatment was initiated, and mice were weighed daily. Mice were treated daily by oral gavage with vehicle control, I-73 (25 mg/kg), lapatinib (100 mg/kg) or a combination thereof. Mice were sacrificed after 5 days of treatment, 24 h after the last treatment, and tumour pieces were snap-frozen. Proteins were isolated from tumour pieces using cold tumour lysis buffer (20 mM Tris-HCL, 137 mM NaCl, 2 mM EDTA, 1% Triton, 10% glycerol in miliQ, freshly supplemented with protease inhibitor cocktail (Sigma; P8340), sodium fluoride (NaF, 10 mM), sodium orthovanadate (NaVO3, 1 mM). The lysate was spun down and supernatant containing proteins were denatured by addition of sample buffer containing 10% β-mercaptoethanol (Acros Organics 125472500). Western blots were performed as described.

Next, an intervention study on tumour growth by these treatments was performed in Hs578T and patient-derived xenograft mouse models. Hs578T cells were xenografted as described for the pharmacodynamics study. For the TNBC patient-derived xenograft (PDX) model, tumour pieces were transplanted into female NOD-Scid IL2Rgnull mice. This PDX model (T250) was established as described previously [64]. Once these tumours reached a size of 200–250 mm^3^, the mice were randomly stratified into one of the treatment arms, the treatment was initiated, and mice were weighed daily. For Hs578T xenografts, 8–11 animals were used per treatment group: vehicle (*n* = 11), lapatinib (*n* = 11), I-73 (*n* = 8) and lapatinib and I-73 combination (*n* = 8). For PDX, the following number of animals were used per treatment group: vehicle (*n* = 5), lapatinib (*n* = 6), I-73 (*n* = 7) and lapatinib and I-73 combination (*n* = 8) were used. Mice were excluded from the experiment in the case of handling errors (e.g. injection in lung), and are not included in the number of mice for each treatment arm. Treatment was performed as described above, until the tumour reached a maximum size of 1500 mm^3^, or for a maximum of 30 days (Hs578T xenograft) or 65 days (PDX). Mice were sacrificed 6 h after the last treatment. Tumour size was measured once every two to three days using a calliper. To calculate mean tumour size for each mouse, measured on different days after the start of the treatment, average tumour sizes for three days were calculated. As some mice reached a tumour size of 1500 mm^3^ earlier than others, the latest measured tumour size (> 1500 mm^3^) was used after sacrification to plot the mean tumour size.

### Data analysis and statistics

All data were processed in GraphPad Prism 7.0 and/or Microsoft Excel. IC_50_ values were derived from GraphPad Prism 7.0 using nonlinear regression fitting. The synergistic effect of drug combinations was analysed by combination index calculations (CI) [65], using the formula CI = D1/D1x + D2/D2x, in which D1 and D2 are the respective combination doses of drug 1 and drug 2 that yield an effect of 50% of proliferation inhibition, and D1x and D2x are the corresponding single doses for drug 1 and drug 2 that result in 50% of proliferation inhibition (i.e. IC_50_). Two-way ANOVA with Tukey’s correction for multiple testing was used to assess impact of drug treatment on cell cycle distribution. One-way ANOVA with Tukey’s correction for multiple testing was used to assess the effect of RNAi-mediated silencing of transcription factors on TNBC cell proliferation. P-values < 0.05 were considered statistically significant.

## Supplementary Information

Below is the link to the electronic supplementary material.

Supplementary Material 1

Supplementary Material 2

Supplementary Material 3

## Data Availability

RNA-sequencing data have been deposited to GEO (GSE283178). Count data is available in Table S1. Uncropped full length western blots are shown in Supplementary Material 2.
