# Supplementary material for: Disrupting CDK9 activity suppresses triple-negative breast cancer and is enhanced by EGFR Inhibition
Source: Cell Oncol (Dordr). 2026 Jan 8;49(1):20. doi: 10.1007/s13402-025-01154-6 (PMC12783313; doi:10.1007/s13402-025-01154-6)
Supplement: Supplementary file 1 — Supplementary Material 1 [file 13402_2025_1154_MOESM1_ESM.docx]

**Supplementary File 1: Supplementary Figures S1-S5**

**Disrupting CDK9 activity suppresses triple-negative breast cancer and is enhanced by EGFR inhibition**

Vera E. van der Noord, Ronan P. McLaughlin, Jessica S. Karuntu, Jichao He, A. Mieke Timmermans, Sunita K. C. Basnet, Yi Long, Sarah Al Haj Diab, Solomon Tadesse, Natalie Proost, Bastiaan van Gerwen, Bjørn Siteur, Marieke van de Ven, Chantal Pont, Sylvia E. Le Dévédec, John W.M. Martens, Shudong Wang, Yinghui Zhang, Bob van de Water


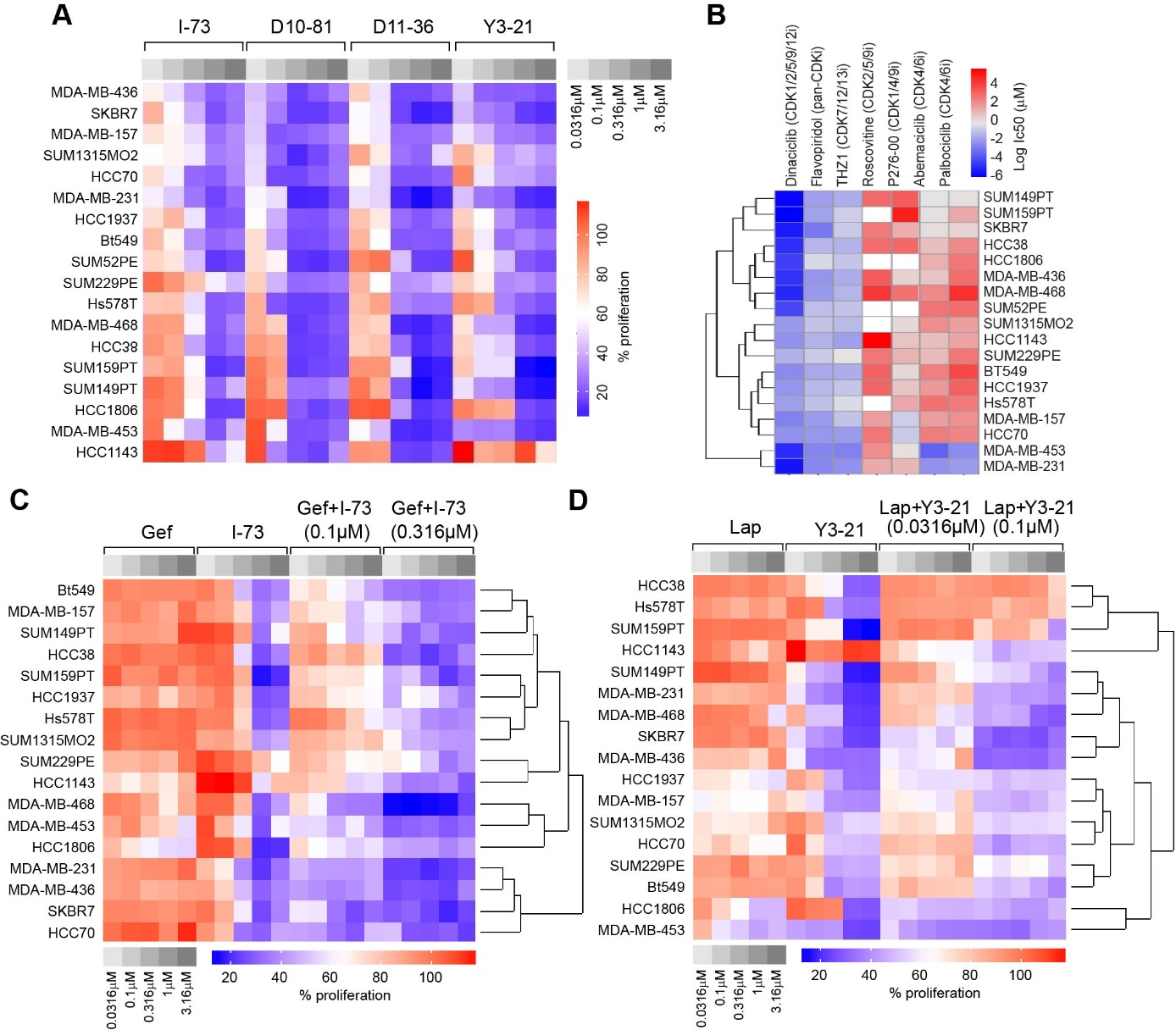


**Fig. S1. CDK9 inhibitors inhibit proliferation of TNBC cells and synergize with EGFR inhibitors.** **(A)** Proliferation of TNBC cell lines after treatment with CDK9 inhibitors I-73, D10-81, D11-36 and Y3-21 at different concentrations (0.0316 µM – 3.16 µM). **(B)** IC50 of CDK inhibitors, including multi-CDK inhibitors dinaciclib, flavopiridol, and roscovitine, CDK7/12/13 inhibitor THZ1, and CDK4/6 inhibitors abemaciclib and palbociclib, to inhibit proliferation in TNBC cell lines. **(C)** Proliferation of TNBC cell lines after treatment with I-73 (0.1 µM and 0.316 µM), a concentration range of gefitinib, or a combination thereof. **(D)** Proliferation of TNBC cell lines after treatment with Y3-21 (0.0316 µM and 0.1 µM), a concentration range of lapatinib, or a combination thereof.


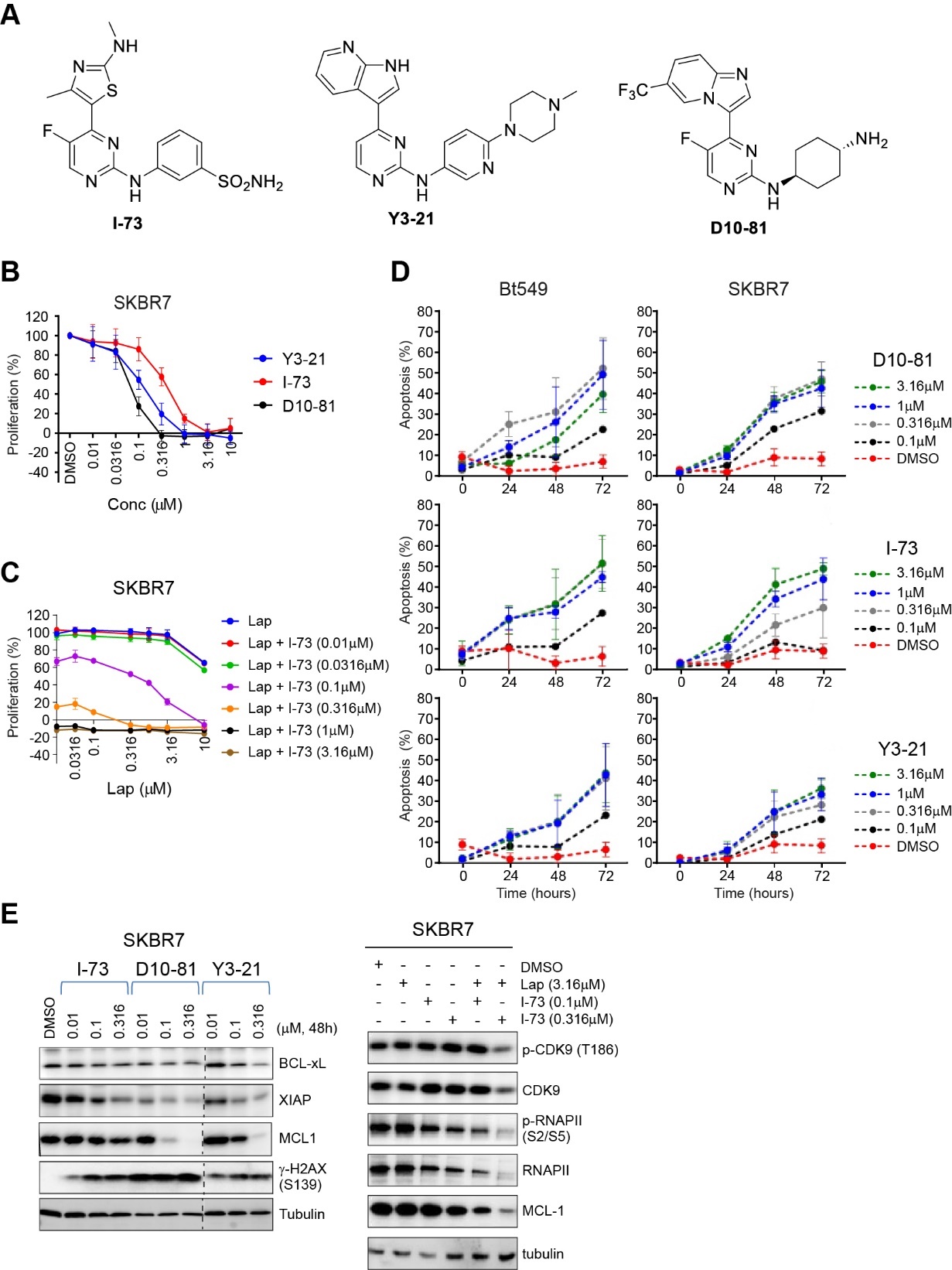


**Fig. S2. CDK9 inhibitors and combination treatment with lapatinib induce apoptosis and inhibit CDK9 downstream targets. (A)** Molecular structure of I-73, Y3-21 and D10-81. **(B)** Dose response curves of concentration range of Y3-21, I-73 and D10-81 in SKBR7 cells. **(C)** Dose response curves of concentration range of lapatinib with a combination of different doses of I-73. **(D)** Induction of apoptosis (% of annexin V positive cells) after treatment with different doses of I-73, D10-81, Y3-21 for 72 hours in Bt549 and SKBR7 cells. **(E)** Effect of different doses of I-73, D10-81, Y3-21 (left) and combination treatment with lapatinib (3.16 µM) and I-73 (0.1 and 0.316 µM) (right) on RNA polymerase II phosphorylation levels, expression of pro-survival proteins BCL-xL, XIAP, MCL1, and H2AX phosphorylation (S139) in SKBR7 cells. Dotted lines are cropmarks for skipped lanes within the same blot.


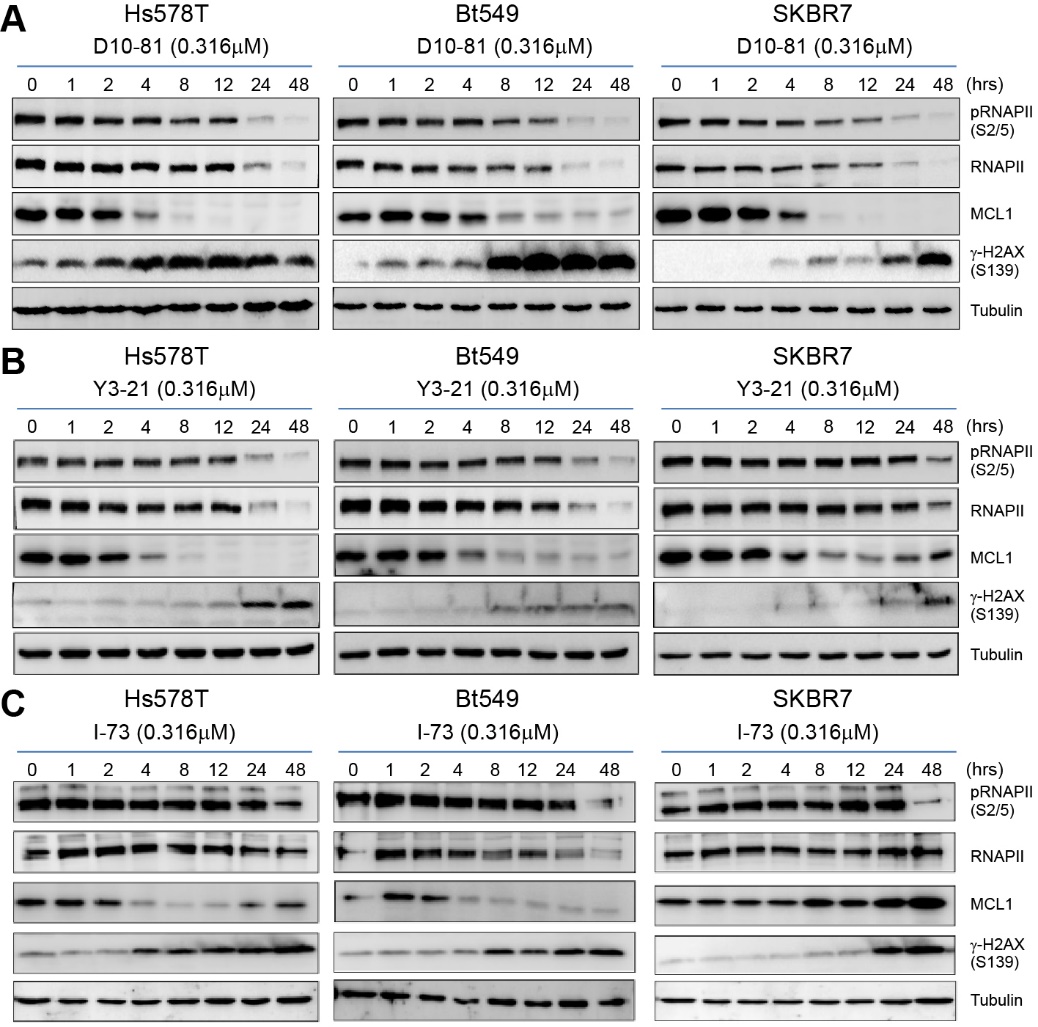


**Fig. S3. Dynamics of P-TEFb complex inhibition, MCL1 downregulation and induction of DNA damage in TNBC cells. (A-C)** Effect of D10-81 **(A)**, Y3-21 **(B)** and I-73 **(C)** on RNA polymerase II (phosphorylation), MCL1 and y-H2AX phosphorylation (S139) protein levels over time (0-48 hours) in Hs578T, BT549 and SKBR7 cells.


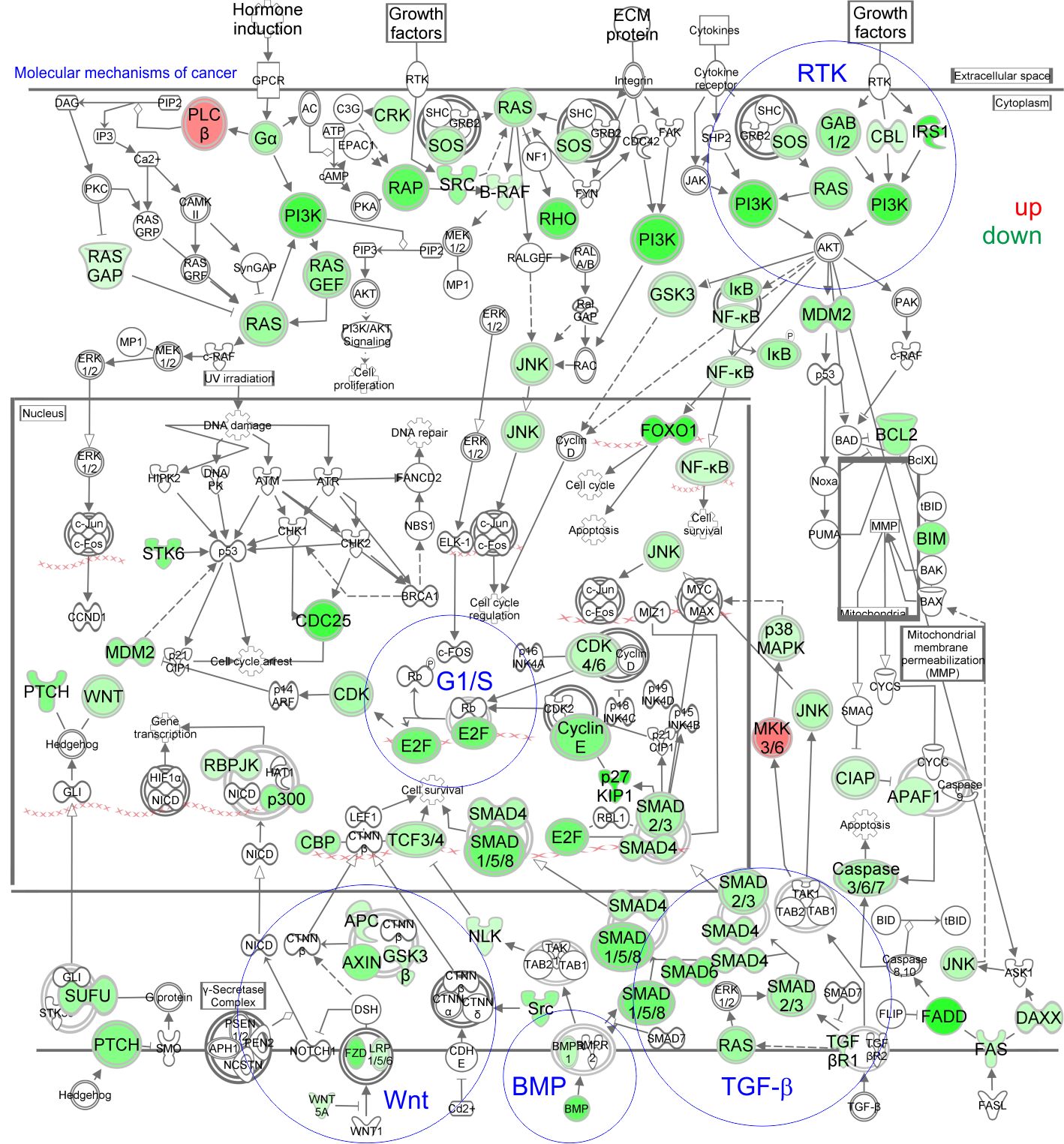


**Fig. S4. Massive disruption of molecular mechanisms of cancer by CDK9 inhibitors.** Ingenuity pathway analysis showing downregulation (green) or upregulation (red) of genes after 6 hours treatment with CDK9 inhibitors in Hs578T cells, as determined by RNA-sequencing. Effect was shown for the CDK9 inhibitor D10-81 representatively.


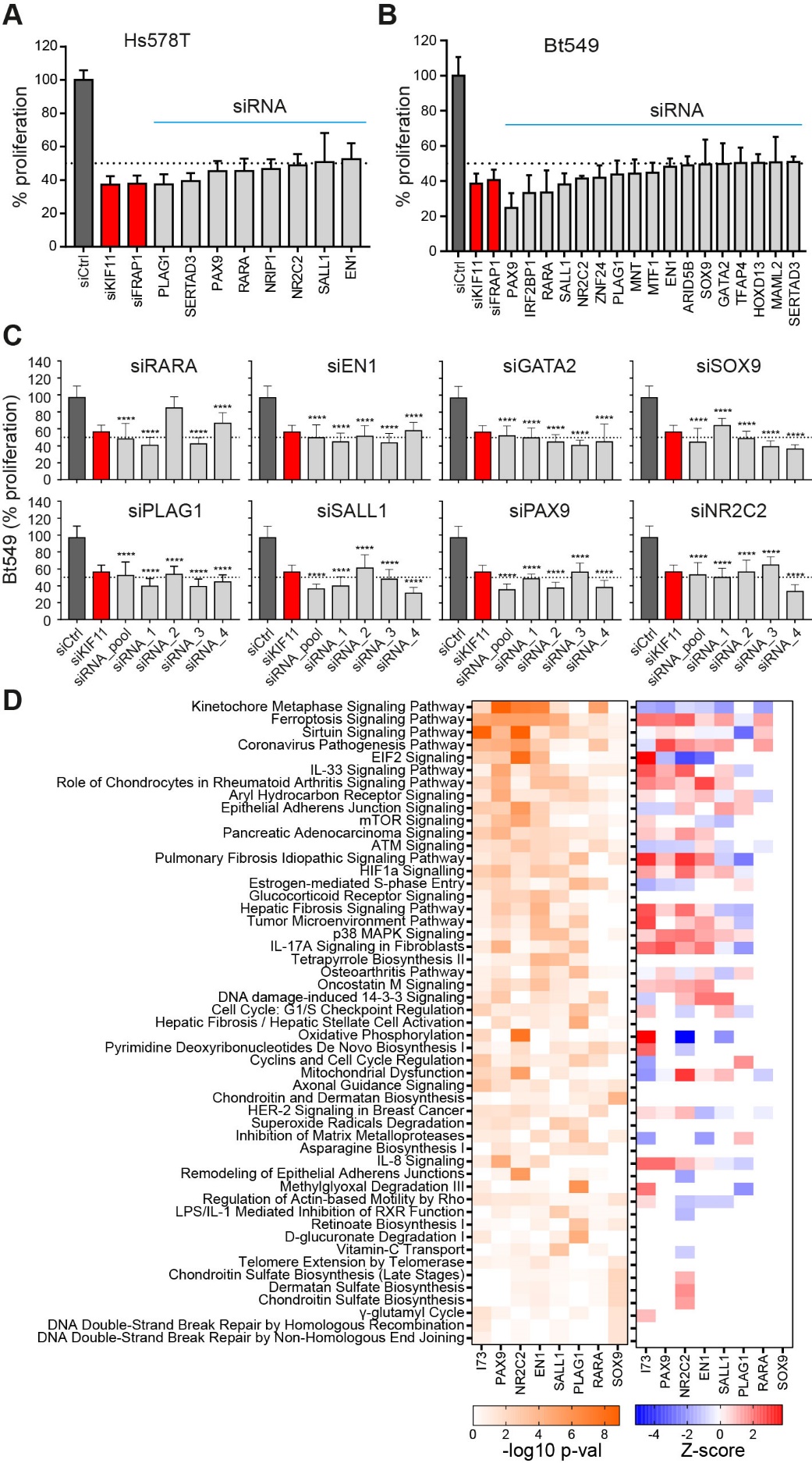


**Fig. S5. CDK9 inhibitors suppress transcription factors essential for proliferation in TNBC cells. (A-B)** Proliferation after knockdown of CDK-inhibitor sensitive transcription factors in Hs578T **(A)** or BT549 **(B)** cells. Genes whose silencing reduced the proliferation by ≥ 50% compared to negative siRNA control (siCtrl) are shown. Positive siRNA controls are shown in red (siKIF11 and siFRAP1). **(C)** SiRNA knockdown of RARA, PLAG1, EN1, SALL1, PAX9, NR2C2, SOX9 using pooled and four individual siRNA sequences in BT549 cells. Data shown as mean ± standard deviation (n = 2, ****=P<0.0001). Results shown as % proliferation normalised to siCtrl. **(D)** Ingenuity pathway analysis on differentially expressed genes (log2FC≥-0.5 or ≥0.5, padj≤0.05) after knockdown of PAX9, NR2C2, EN1, SALL1, PLAG1, RARA and SOX9. Shown are p-value (-log10, left) and Z-score (right) of the top 10 (based on p-value) pathways for each knockdown.
