# Supplementary material for: Disrupting CDK9 activity suppresses triple-negative breast cancer and is enhanced by EGFR Inhibition
Source: Cell Oncol (Dordr). 2026 Jan 8;49(1):20. doi: 10.1007/s13402-025-01154-6 (PMC12783313; doi:10.1007/s13402-025-01154-6)

**Supplementary Material 2. Uncropped western blot images**

**Disrupting CDK9 activity suppresses triple-negative breast cancer and is enhanced by EGFR inhibition**

Vera E. van der Noord, Ronan P. McLaughlin, Jessica S. Karuntu, Jichao He, A. Mieke Timmermans, Sunita K. C. Basnet, Yi Long, Sarah Al Haj Diab, Solomon Tadesse, Natalie Proost, Bastiaan van Gerwen, Bjørn Siteur, Marieke van de Ven, Chantal Pont, Sylvia E. Le Dévédec, John W.M. Martens, Shudong Wang, Yinghui Zhang, Bob van de Water

**
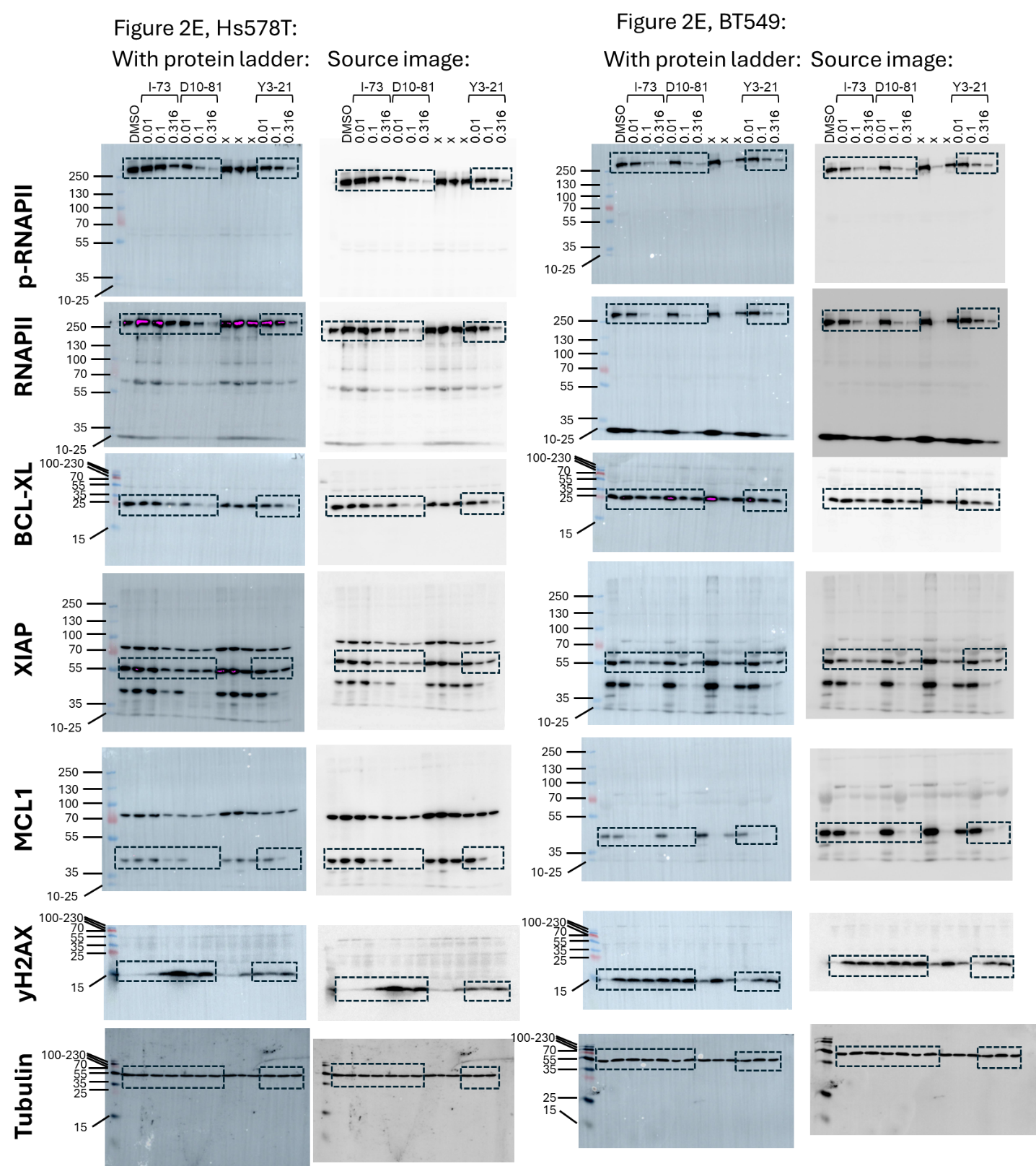
**


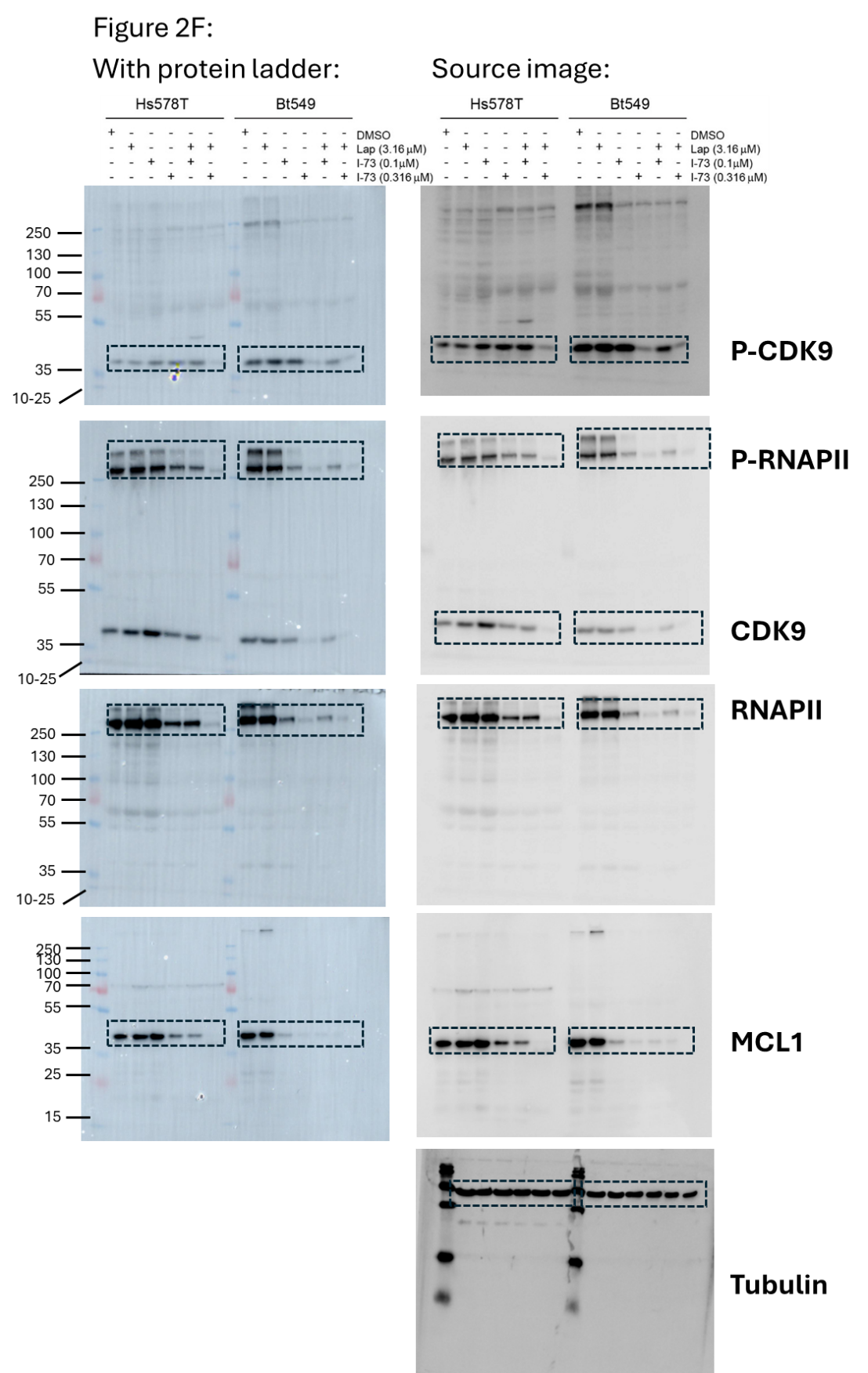


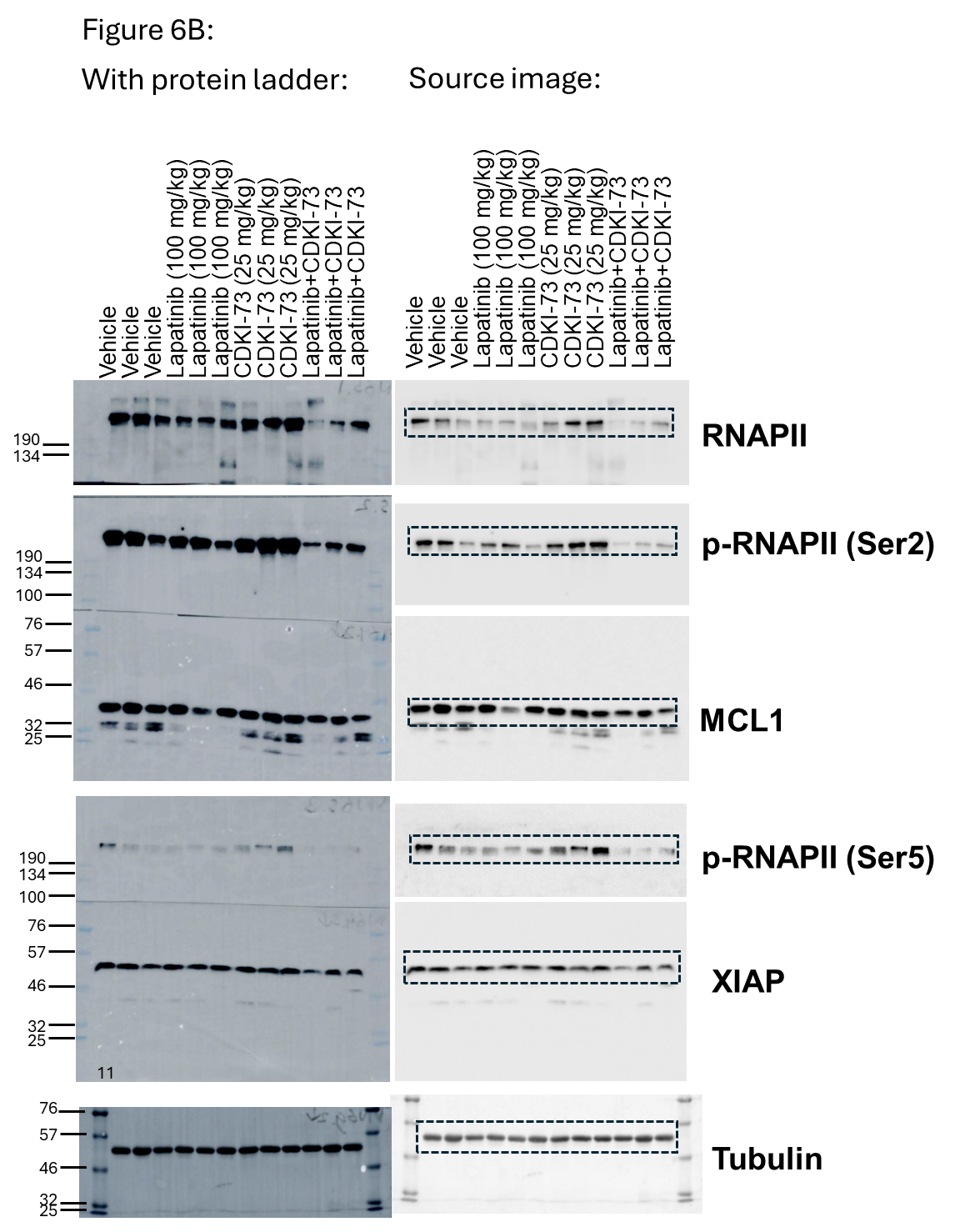

Supplement: Supplementary file 2 — Supplementary Material 2 [file 13402_2025_1154_MOESM2_ESM.docx]
